# Durability and Surface Oxidation States of Antiviral Nano-Columnar Copper Thin Films

**DOI:** 10.1021/acsami.3c01400

**Published:** 2023-03-22

**Authors:** Keisuke Shigetoh, Rie Hirao, Nobuhiro Ishida

**Affiliations:** Toyota Central R&D Labs., Inc., 41-1 Yokomichi, Nagakute, Aichi 480-1192, Japan

**Keywords:** antiviral activity, SARS-CoV-2, COVID-19, antiviral coatings, copper, nanostructured thin films, oxidation state

## Abstract

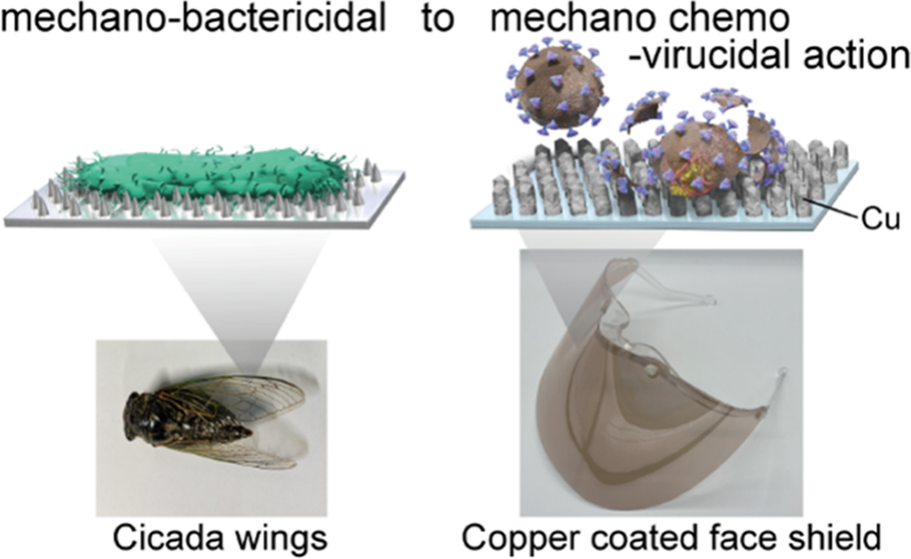

Antiviral coatings that inactivate a broad spectrum of viruses are important in combating the evolution and emergence of viruses. In this study, nano-columnar Cu thin films have been proposed, inspired by cicada wings (which exhibit mechano-bactericidal activity). Nano-columnar thin films of Cu and its oxides were fabricated by the sputtering method, and their antiviral activities were evaluated against envelope-type bacteriophage Φ6 and non-envelope-type bacteriophage Qβ. Among all of the fabricated films, Cu thin films showed the highest antiviral activity. The infectious activity of the bacteriophages was reduced by 5 orders of magnitude within 30 min by the Cu thin films, by 3 orders of magnitude by the Cu_2_O thin films, and by less than 1 order of magnitude by the CuO thin films. After exposure to ambient air for 1 month, the antiviral activity of the Cu_2_O thin film decreased by 1 order of magnitude; the Cu thin films consistently maintained a higher antiviral activity than the Cu_2_O thin films. Subsequently, the surface oxidation states of the thin films were analyzed by X-ray photoelectron spectroscopy; Cu thin films exhibited slower oxidation to the CuO than Cu_2_O thin films. This oxidation resistance could be a characteristic property of nanostructured Cu fabricated by the sputtering method. Finally, the antiviral activity of the nano-columnar Cu thin films against infectious viruses in humans was demonstrated by the binding inhibition of the SARS-CoV-2 spike protein to the angiotensin-converting enzyme 2 receptor within 10 min.

## Introduction

The COVID-19 pandemic caused by the SARS-CoV-2 virus and its variants continues to exert a significant impact on our health and economic activities to date, despite the administration of more than 12 billion vaccine doses worldwide (till September 2022).^1,2^ Close contact, respiratory-droplet inhalation, indirect transmission via contaminant surfaces, and airborne transmission via aerosol are the major potential human-infection routes.^3^ The virus remains viable and infectious in aerosols for hours and on surfaces for numerous days, increasing the significance of indirect transmission routes.^3,4^ Furthermore, the vaccine administered in the initial stages of the pandemic exhibits lower efficacy against SARS-CoV-2 variants.^5^ Therefore, to combat the rapid evolution and emergence of viruses, it is vital to develop antiviral coatings for virus inactivation that can be used in combination with vaccination.

Optimizing the material selection (including its chemical state) and coating conditions (effective surface area, transparency, flexibility, stability, etc.) is vital for maximizing the antiviral-coating activity and practicability. In this study, copper was selected as the material due to the following reasons. First, copper compounds possess antibacterial activity (known since 2600 B.C.) and have been utilized to sterilize wounds and drinking water.^6^ Second, copper compounds show a broad spectrum of antiviral activity against both envelope- and non-envelope-type viruses.^7^ The inactivation mechanisms of copper compounds include the generation of reactive oxygen species by leached copper ions, surface catalysis or contact killing, and disulfide bond breakage of viral proteins;^3,7−10^ the last two mechanisms are particularly important.^7,8^ Reduction of the disulfide bonds in the spike receptor-binding domain (RBD) of SARS-CoV-2 decreases its binding affinity to the angiotensin-converting enzyme 2 (ACE2) by 2 orders of magnitude.^11^ In addition to their significant broad-spectrum antiviral activity, the relatively low cost of copper facilitates practical applications. There are numerous publications reporting Cu-based antiviral coatings,^12−18^ including recent reports on the inactivation of SARS-CoV-2 by Cu nanoparticles,^19^ Cu_2_O,^12,16^ and CuO^13,20^ coatings.

**Figure 1:**
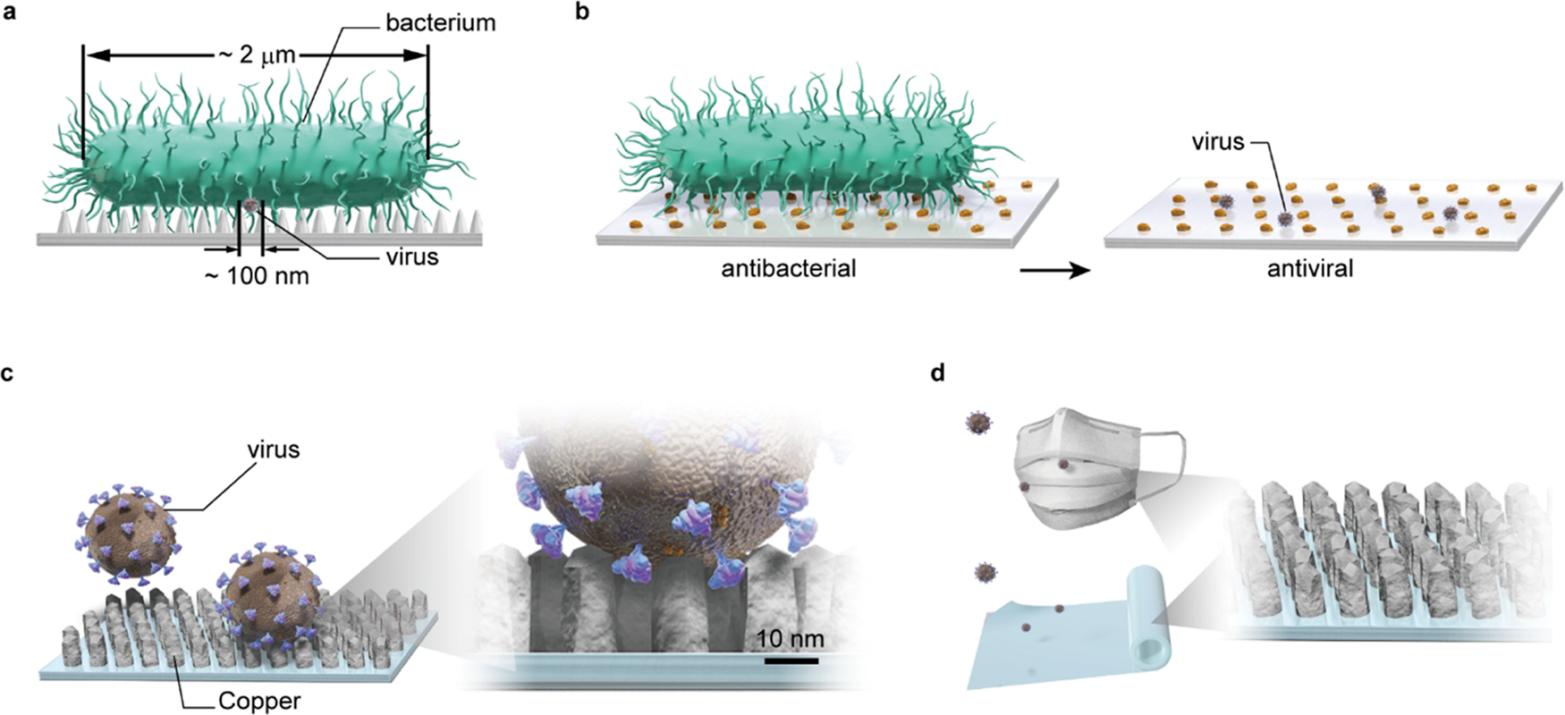
Proposed antiviral nano-columnar Cu coatings to overcome the differences in scale between bacteria and viruses. (a) Vertical schematic view of a bacterium and virus on the cicada-wing-type column-structure surface. (b) Difference in contact efficiency between bacteria and viruses on the surface-dispersed antiviral nanoparticles (orange). (c) Proposed nano-columnar (gray) structure. The diameter of the column is ∼10 nm, which is smaller than the virus, and densely arranged on the surface, maintaining light transparency. (d) Antiviral nano-columnar copper coatings could be applied on disposable face masks and flexible polymer sheets.

Furthermore, an ideal Cu-material coating condition, inspired by cicada wings (with dense columns on the surface) (Figure S1), was developed here. The dense columnar structures on the surface of insects like cicadas and dragonflies kill bacteria via physico-mechanical interactions between the nanostructured surfaces and bacteria.^21−23^ Additionally, surface columns with lower pitch and diameters exhibit higher bactericidal properties.^21^ Therefore, an increased frequency of contact between the virus and copper should enhance viral inactivation by surface catalysis and contact killing. Notably, a scale gap exists while adapting this strategy for viruses. As illustrated in Figure 1a, the cicada wing column widths are ∼200 nm (Figure S1), which are effective against ∼2 μm long bacteria. The column diameter should be lesser than the size of the target to be inactivated. As viruses are about one-twentieth the size (in the longitudinal direction) of bacteria in general, column widths of ∼10 nm are required for virus inactivation. To resolve this scaling problem, several nanostructure coating models have been proposed for next-generation antiviral surface coatings.^3,9^ Additionally, to maintain a high contact frequency between Cu and viruses that are smaller than bacteria, a high coverage rate and small material loading are required, as shown in Figure 1b. Therefore, nano-columnar structured thin copper coatings have been proposed here, as shown in Figure 1c. In this structure, the nano-column width is comparable to the surface spike protein size (∼10 nm) of SARS-CoV-2^24^ (these spike proteins play an important role in the infection).^11^ Notably, the aforementioned coating structure could be fabricated using a low material loading on the polymer surface, ensuring transparency, flexibility, and low environmental risk, thereby facilitating the coating of disposable face masks, filters, and sheets (Figure 1d).

Other than the concept of the copper coating structure, the chemical state of copper is also important. Cuprous oxide (Cu_2_O) shows 3–5 orders of magnitude higher antiviral activity against envelope and non-envelope viruses than cupric oxide (CuO).^7^ Therefore, here, the surface oxidation state of copper was analyzed in addition to its nanostructure. A detailed understanding of the relationship between the surface oxidation state of copper and its antiviral activity is important for designing antiviral coatings and estimating their stability in air. There are no previous reports on the comparison of the antiviral activities of metallic copper (Cu) and its oxides under well-defined conditions. Thus, this paper, containing a detailed analysis of the influence of the nanostructure and chemical state of copper on viral inactivation, is the first to elucidate the novel concept of mechano-chemo-virucidal action.

Here, the antiviral activity of well-defined nano-columnar (10–20 nm) thin films of Cu, Cu_2_O, and CuO, fabricated by the sputtering method, against the model viral bacteriophages Φ6 and Qβ has been investigated. Additionally, the fabricated nano-columnar Cu thin films inhibited the specific binding of the SARS-CoV-2 spike protein to the angiotensin-converting enzyme 2 (ACE2) receptor.

## Materials and Methods

### Materials

A pure Cu target (purity > 99.99%; 50 mm in diameter; Toshima Co. Ltd.) was used to fabricate the thin films by a radio frequency (RF) sputtering method. A Cu plate (purity > 99.96%; 0.5 mm in thickness; Nilako Corp.) was used as the control sample for antiviral activity and oxidation state analyses. The bacteria and bacteriophages Φ6 (NBRC 105899) and Qβ (NBRC 20012) with *Escherichia coli* (NBRC 106373) and *Pseudomonas syringae* (NBRC 14084), respectively, as the host strains were purchased from the NITE Biological Resource Center, NBRC. Bovine serum albumin (#019-27051; FUJIFILM Wako Pure Chemical) was used as a pseudo-contaminating protein.

### Thin Film Fabrication

Thin films of copper (Cu) and its oxides were fabricated using the RF sputtering method with a pure Cu target. The Cu, Cu_2_O, and CuO thin films were deposited in 5-Pa Ar (purity 99.9999%), 3-Pa O_2_ 2%-Ar 98%, and 5-Pa O_2_ 10%-Ar 90% atmospheres, respectively, at an RF power of 100 W. The amount of material deposited during the process was monitored by a quartz crystal oscillator placed near the sample stage and maintained at 5 μg cm^–2^ by regulating a mechanical shutter. Polypropylene (PP) sheets (10 mm × 10 mm or 10 mm Φ, 0.2-mm thickness) and Si wafers (0.4-mm thickness) were used as substrates. The distance between the target and sample stage was 110 mm.

The fabricated samples were exposed to the atmosphere for different air-exposure times (ranging from 1 day to 1 month) by placing them in unsealed containers at room temperature (∼25 °C) and ∼40% relative humidity. The initial condition of the films, 30 min prior to air exposure, was labeled pristine.

### Characterization of the Fabricated Thin Films

#### Crystalline Phases, Mass Loading, and Structural Analyses

The crystalline phases of the fabricated thin films on glass were characterized by X-ray diffraction (XRD) analysis using an Ultima IV (Rigaku) with Cu Kα radiation. For XRD analysis, the deposition amount was maintained in the range of approximately 50–100 μg cm^–2^ to maximize the diffraction intensity. Inductively coupled plasma-atomic emission spectrometry (ICP-AES) by a PS3520VDDII (Hitachi High-Tech Science) instrument was used to estimate the material mass loading of the fabricated thin films. The samples were deposited on Si substrates (cleaved to a size of ∼4 cm^2^) under the aforementioned conditions. The actual geometrical surface area of the Si substrate was determined by image analysis using ImageJ.^25^ The samples were completely dissolved in dilute nitric acid and hydrogen peroxide before ICP-AES measurements. Field-emission scanning electron microscopy (S5500; HITACHI High-Tech) was used to examine the morphology and thickness of the fabricated thin films. The film surfaces were examined by atomic force microscopy (AFM) in the tapping mode using a Nanoscope V (Bruker) instrument; AFM data were analyzed using the Gwyddion software.

#### Determining the Chemical State

A PHI Quantera II (ULVAC-PHI) instrument with a monochromatic Al Kα (1486.6 eV) X-ray source was used for X-ray photoelectron spectroscopy (XPS). Survey spectra and high-energy resolution spectra of Cu 2p, O 1s, and C 1s and X-ray excited Auger peak spectra were collected using pass energies of 280 and 26 eV, respectively, at a take-off angle of 45°. The MultiPak software (version 9.9.3, ULVAC-PHI) was used for spectral analysis, after the Shirley-type background subtraction, followed by peak deconvolution considering the known binding energies of the related species.^26−30^ The energy shift of the XPS spectra due to the charging effect was corrected using the C 1s peak of the adventitious carbon at a binding energy of 284.8 eV. Peak areas of the obtained spectrum in the Cu 2p region (925–970 eV) after the Shirley-type background subtraction were used as normalization constants to analyze the spectral intensity of measured regions. The Cu 2p_3/2_ region was used for the peak deconvolution of the Cu 2p region. The metallic Cu [Cu(0)] state can be distinguished from the oxide Cu_2_O [Cu(I)] state using Cu LMM Auger spectrum analysis, due to higher energy shifts in the Cu LMM Auger spectrum compared to those in the Cu 2p spectrum.^31^ The procedure of Cu LMM spectrum deconvolution is outlined in previous publications.^30^ Reconstructed functions of the decomposed Cu LMM spectrum were used to obtain the spectra of standard samples using five peaks as fitting functions (Supplementally Note 1, Figure S2). The area fractions of the Cu species obtained by spectrum deconvolution are summarized in Table S1. The relative fraction of Cu species in each spectrum was calculated using the ratio of their peak areas. In principle, the Cu_2_O and Cu(OH)_2_ states should be indistinguishable in the Cu LMM spectrum. Therefore, only the relative fraction of metallic Cu could be evaluated from the ratio of Cu: (Cu_2_O + Cu(OH)_2_): CuO, which was calculated using the area fractions estimated by the standard fitting functions of Cu, Cu_2_O, and CuO (Figure S2). To quantify the relative fractions of Cu_2_O, CuO, and Cu(OH)_2_ on the sample surface, a combination of signals from the main Cu 2p_3/2_ peaks of Cu + Cu_2_O, CuO, Cu(OH)_2_ and the satellite peaks (at binding energies of ∼7.5 and 10 eV higher than that of the main peak) were used.^32^ The Cu 2p_3/2_ region was deconvoluted to determine the final existence ratio of CuO and Cu(OH)_2_. The Cu_2_O fraction was determined by subtracting the Cu fraction obtained from the Cu LMM region from the Cu + Cu_2_O fraction obtained from the deconvolution of the Cu 2p_3/2_ region.

#### Oxidation Layer Structure of the Fabricated Thin Films

To predict the oxidation layer structure of the fabricated Cu thin films along the thickness direction, the oxide film thickness (*d*_ox_) was estimated using the Strohmeier equation^31,33^
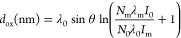
1

A take-off angle of 45° was used for θ in eq 1, and the ratio of the volume density of copper atoms in bulk copper to those in the oxide (*N*_m_/*N*_o_) was 1.68 for Cu_2_O and 1.77 for CuO (calculated from the ratio of the number of copper atoms in the unit cell volume). The inelastic mean free pass values, λ_o_ (Cu_2_O) = 2.84, λ_o_ (CuO) = 2.76, and λ_m_ (Cu) = 1.36 were calculated using Seah and Dench’s empirical eqs 2 and 3.^34^
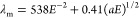
2
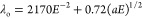
3

In eqs 2 and 3, *a* is the monolayer thickness (*a*_Cu_ = 0.012 nm, *a*_Cu_2_O_ = 0.017 nm, and *a*_CuO_ = 0.016 nm^31^) and *E* is the kinetic energy. The Cu LMM X-ray excited Auger peaks (Cu = 918.6 eV, Cu_2_O = 916.2 eV, CuO = 918.1 eV^31^) were used for *E*. The peak area ratios (*S*_o_/*S*_m_) obtained from the Cu LMM spectra were used as the intensity ratios (*I*_o_/*I*_m_) in eq 1.

### Characterization and Inactivation of Bacteriophages

#### Preparation and Purification of Viral Suspensions

Two types of viruses with different surface structures (the envelope-type bacteriophage Φ6 and non-envelope-type bacteriophage Qβ) were used as model viruses. A lysogeny broth (LB) (Formedium) medium containing calcium chloride (2 mM) (Ca-added LB medium) was used to prepare their stock suspensions. The respective bacteriophages were infected after incubation at 37 and 30 °C for *E. coli* and *P. syringae*, respectively, until the logarithmic growth phase. For antiviral activity analyses, the prepared stock suspensions were purified and concentrated using an ultrafiltration device (Amicon Ultra-4,10 kDa, Merck). The plaque assay, a common method for evaluating bacteriophages, was used to analyze the viral infection titer.^8^ The viral titer was estimated using the plaque-forming units (PFU). Culture plates were prepared by adding 1.5% (wt vol^–1^) agar powder (FUJIFILM Wako Pure Chemical) into the Ca-added LB medium; additionally, 0.6% (wt vol^–1^) agar powder was added to the Ca-added LB medium as a top agar for the plaque assay. The viral suspension purity (PFU mg_-protein_^–1^) is defined as the viral titer concentration of the stock suspension divided by the protein concentration [(PFU mL^–1^)/(mg_-protein_ mL^–1^)]. The protein concentrations of the stock suspensions were determined by measuring their absorbance at 280 nm using a NanoDrop spectrophotometer (ND-1000, Thermo Fisher Scientific).

#### Morphology Observation

The transmission electron microscope (TEM) analysis of the bacteriophages was performed using a JEM1400Flash electron microscope (JEOL) at 100 kV. Viral suspensions of the bacteriophages (Φ6 and Qβ) were applied to the carbon-coated TEM grids. Subsequently, after partial drying, they were stained with 2% uranyl acetate for 10 s before analysis.

#### Bacteriophage Inactivation

The purities of the viral stock suspensions of the bacteriophages Φ6 and Qβ used for the antiviral effect analysis were 1 × 10^11^ PFU mg_-protein_^–1^ and in the range from 7 × 10^11^ to 4 × 10^12^ PFU mg_-protein_^–1^, respectively. Before experimentation, their stock suspensions were adjusted using a 1/500 NB buffer solution (500 times diluted NB buffer in Milli-Q water) to a final concentration of ∼1.7 × 10^9^ PFU mL^–1^. Subsequently, 6 μL (*N*_o_ ∼ 1 × 10^7^ PFU sample^–1^) of the diluted suspensions was sandwiched between the samples (10 mm × 10 mm specimens) and transparent thin plastic films (8 mm × 8 mm) to maximize contact and avoid drying; the contact of the sample with the viral solution was allowed to proceed in the dark. The illuminance near the samples during the test, measured using a light analyzer (LA-105, NK system), was ∼10 Lux. After a predetermined contact time, the specimens were washed with a 1/500 NB buffer solution, and the virus solutions were collected. The collected washings were mixed with the log-phase host culture medium to infect the host at 37 and 25 °C for the bacteriophages Qβ and Φ6, respectively, for 5 min. Subsequently, the infected solution (mixed solution of bacteriophage and host culture medium) was spread on the bottom-agar-filled plates, together with the top agar, followed by incubation at 37 °C (for 16 h) and 25 °C (for 40 h) for the bacteriophages Qβ and Φ6, respectively. The number of appearing plaques, *N* (PFU), was counted using a colony counter Scan 500 (Interscience) instrument (Figure S3). The antiviral activities of the samples were evaluated by the log reduction of the viral titer, defined as log_10_(*N*/*N*_0_). The frequency of no plaque formation (below the detection limit) was also used as a reference for its antiviral activity. After experimentation with several specimens, the average values of log_10_(*N*/*N*_0_) were plotted with error bars of the standard deviation of data. At measurement points where data were available both below and above the detection limit, the average values and standard deviation were calculated by uniformly setting log_10_(*N*/*N*_0_) = −5.4 for the case below the detection limit.

### SARS-CoV-2 Spike-ACE2 Binding Assay

The inhibiting abilities of the nano-columnar Cu thin films for the binding of the spike S1 protein (spike protein) to ACE2 were investigated using a commercially available ACE2: SARS-CoV-2 spike S1 inhibitor screening assay kit (BPS Bioscience, Cat. #79945), while a GloMax-Multi Detection System (Promega) was used for chemiluminescence analysis. Cu thin films fabricated on a 10-mm-diameter PP substrate and the bare PP substrate (as a control sample) were placed at the bottom of a 48-well plate, and 200 μL of the spike protein solution with a concentration of 1 ng μL^–1^ (10 nM) was poured on them. The plate was gently shaken for the reaction to proceed for the prescribed time (10–40 min), with a seal covering it to minimize solution drying. Subsequently, the solution (50 μL) collected from each well was introduced into the ACE2-coated 96-well plate. After some adjustments according to the manufacturers’ manual, the luminescence intensity was measured, and spike protein solutions of several known concentrations (0.12, 0.37. 1.1, 3.3, 10, and 20 nM) were used to plot a concentration calibration curve (Figure S4).

## Results and Discussion

### Crystalline Phase Analysis of the Fabricated Thin Films

**Figure 2:**
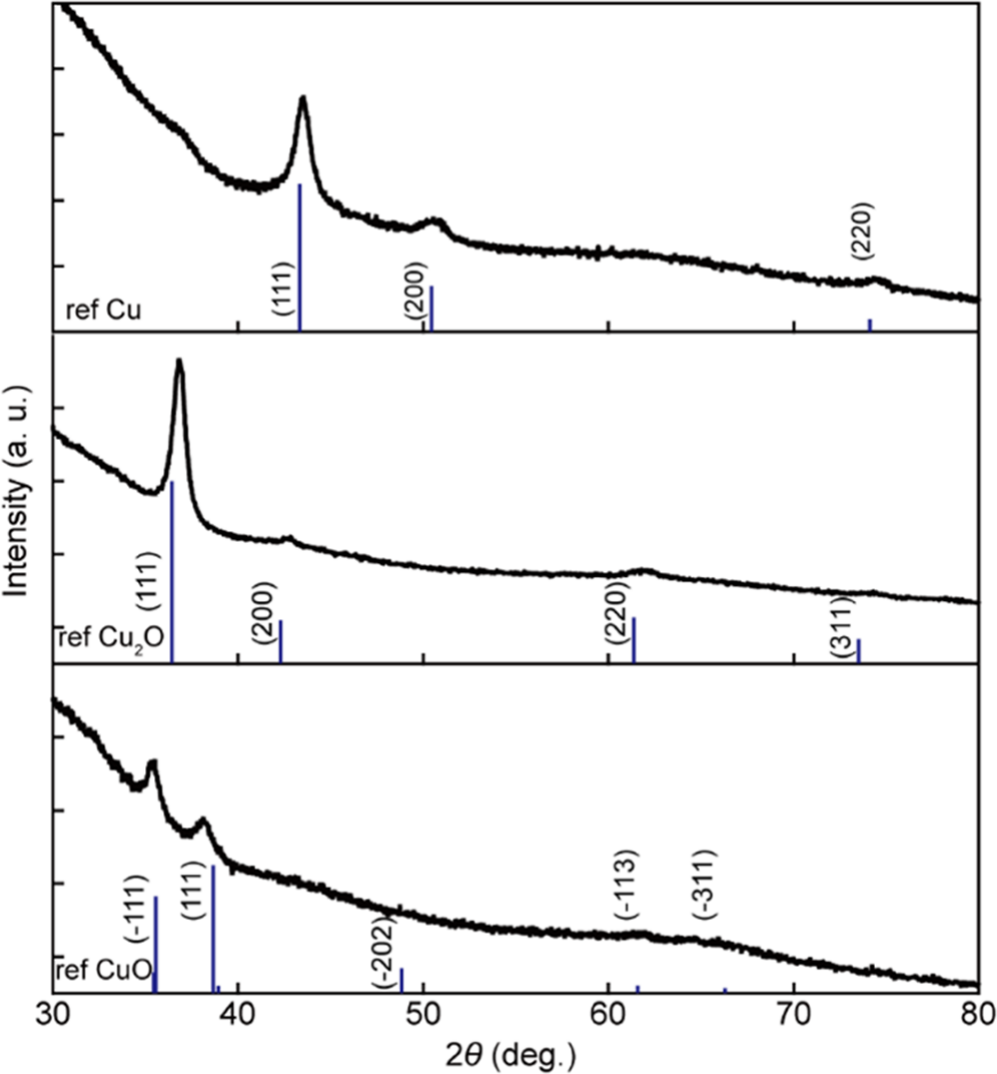
Crystalline phases of the fabricated thin films. The XRD patterns of the fabricated thin films, deposited on glass under 5-Pa Ar 100% (top), 3-Pa O_2_ 2%-Ar 98% (middle), and 5-Pa O_2_ 10%-Ar 90% (bottom) are shown, with the reference peak positions and intensities of Cu (ICDD PDF No. 98-000-0172), Cu_2_O (ICDD PDF No. 98-000-0186), and CuO (ICDD PDF No. 04-012-7238), respectively, indicated by vertical blue lines.

The crystal phases of the fabricated thin films were characterized by XRD, as shown in Figure 2. The XRD peaks of the thin films deposited under 5-Pa Ar 100% (Figure 2, top), 3-Pa O_2_ 2%-Ar 98% (Figure 2, middle), and 5-Pa O_2_ 10%-Ar 90% (Figure 2, bottom) are shown with the reference peak positions and intensities of Cu (ICDD PDF No. 98-000-0172), Cu_2_O (ICDD PDF No. 98-000-0186), and CuO (ICDD PDF No. 04-012-7238), respectively, by vertical lines. All of the peaks are consistent with the reference peaks, except for a shoulder at around 2θ = 36.4° in Figure 2 (top); this shoulder indicates a small amount of Cu_2_O in the Cu thin film. No significant deviation in intensities of specific lattice planes (*hkl*) relative to the intensity ratios in the reference XRD data was observed, indicating the absence of uniaxially oriented crystals. Therefore, the Cu, Cu_2_O, and CuO thin films fabricated by the sputtering method were polycrystalline, with almost a single phase. Thus, the thin films fabricated under 5-Pa Ar 100%, 3-Pa O_2_ 2%-Ar 98%, and 5-Pa O_2_ 10%-Ar 90% were labeled Cu, Cu_2_O, and CuO thin films, respectively.

The mass loading values of the fabricated thin films (6.2, 5.9, and 5.7 μg_-Cu_ cm^–2^ for Cu, Cu_2_O, and CuO, respectively) were confirmed by ICP-AES. These values were in good agreement with the mass loading values set using a quartz crystal oscillator during the thin film deposition process (5 μg cm^–2^).

### Structural Characterization of the Fabricated Thin Films

A cross-sectional FE-SEM analysis of the thin films after cleaving the samples and Si substrate was used to evaluate their thicknesses. The Cu, Cu_2_O, and CuO thin films were 13, 14, and 20 nm thick, respectively. Their top, bird’s-eye, and cross-sectional views are shown in Figure S5. All of the thin films showed densely arranged columnar structures 5–10 nm in width.

**Figure 3:**
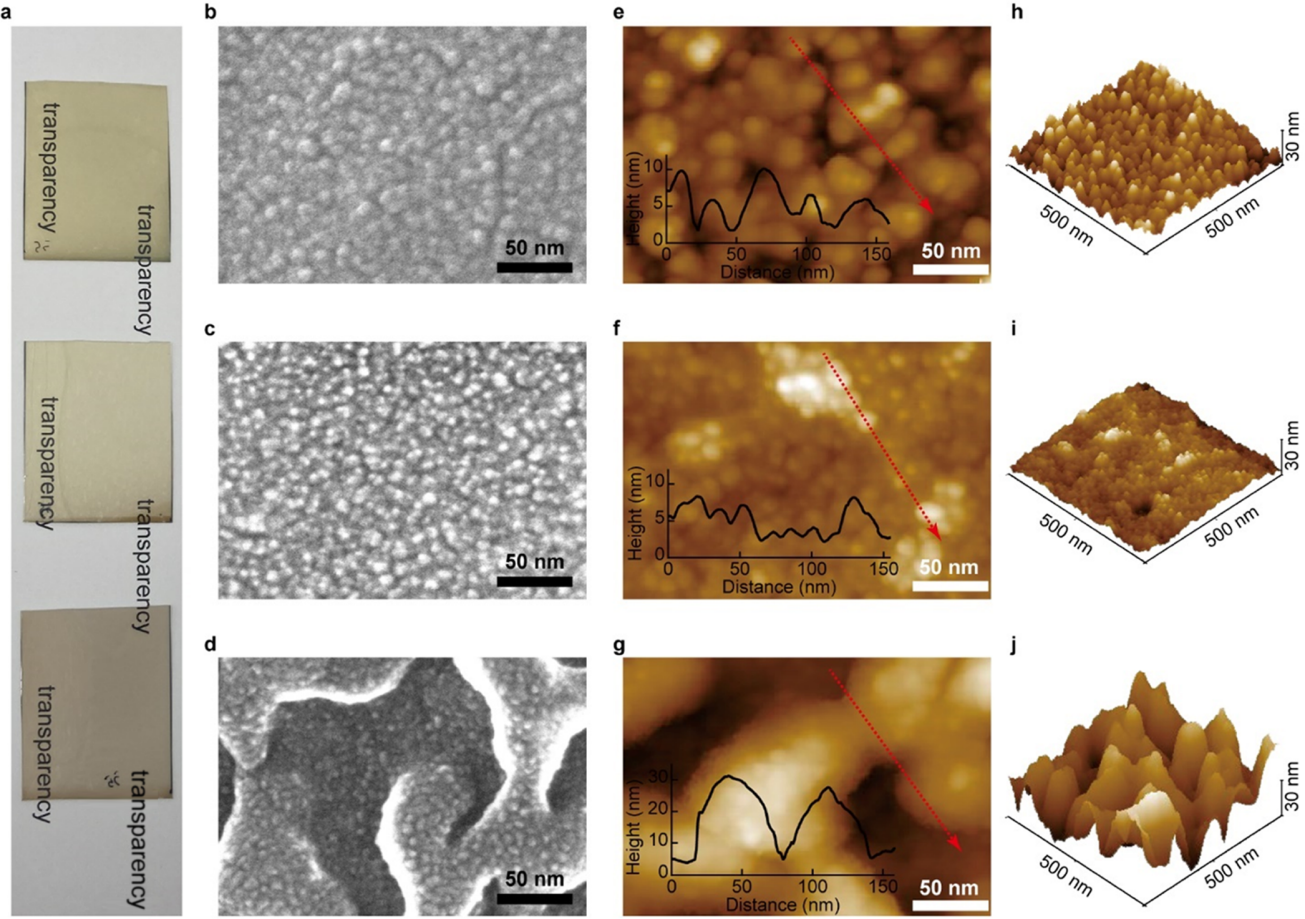
Photographs and structures of the transparent polymer films with nano-columnar Cu and Cu-oxide coatings. (a) Photographs of the fabricated Cu (top), Cu_2_O (middle), and CuO (bottom) thin films on a polypropylene (PP) substrate (50 mm × 40 mm). FE-SEM top views of the (b) Cu, (c) Cu_2_O, and (d) CuO thin films on the PP substrate. AFM images of the (e) Cu, (f) Cu_2_O, and (g) CuO thin films on the PP substrate. The insets show the line profiles along the direction of the red arrows. 3D AFM views (500 nm × 500 nm) of the (h) Cu, (i) Cu_2_O, and (j) CuO thin films on the PP substrate. The scales of height are 30 nm.

Figure 3a shows photographs of the Cu, Cu_2_O, and CuO thin films on the PP substrate. All of the thin films were colored and transparent. Figure 3b–d shows the top views of the Cu, Cu_2_O, and CuO thin films, respectively, on the PP substrate, as indicated by the FE-SEM analysis. Although sample charge-up (due to less conductive PP substrates below the thin films) made it challenging to record a clear image, compared to the case for films on Si substrate (Figure S5), a nano-size column arrangement was confirmed. Notably, in the CuO thin film (Figure 3d), a secondary structure of nano-size columns was observed on the surface of the primary structure; it was raised and exhibited a width of ∼50 nm. This raised structure was not observed on the CuO thin film fabricated on the Si substrate (Figure S5); thus, it could be attributed to the surface-energy difference due to different substrates.

As shown in the top-view AFM image of the Cu thin film (Figure 3e), the diameter of the Cu column was 10–20 nm, which is larger than that observed in the FE-SEM image (Figure 3b). This could be because the AFM Si probe (curvature radius ∼7 nm) could not sufficiently penetrate the gap between the columns and was unable to distinguish between neighboring columns. A line-scan result of the height along the red arrow (distance *l* ≈ 150 nm) is included in the figure; the height difference between the peaks and valleys of the columns was ∼8 nm. *R*_z_ (*l* ≈ 700 nm), calculated as a statistical value, was 6.2 nm (Figure S6a). However, the height difference lacks accuracy because the tip of the probe could not sufficiently penetrate the space between the columns. Figure 3h shows an AFM 3D view of a 500-nm-square range; it confirms a densely arranged Cu columnar structure. As indicated by the top-view AFM image of the Cu_2_O thin film (Figure 3f), the Cu_2_O column exhibited a diameter of 10–20 nm, which is larger than that observed in the FE-SEM image (Figure 3c). A line-scan result of the height along the red arrow (*l ≈* 150 nm) is included in the figure. The height difference between the peaks and valleys of the columns was ∼3 nm. *R_z_* (*l* ≈ 700 nm), calculated as a statistical value, was 3.1 nm (Figure S6b). Figure 3i shows an AFM 3D view of a 500-nm-square range and confirms a densely arranged Cu_2_O columnar structure; although this structure was sharper, it was lower than that of the Cu thin film. The AFM image of the CuO thin film (Figure 3g) indicated a primary structure with 50-nm width and 30-nm height, consistent with the FE-SEM observations (Figure 3d). Here, unlike the FE-SEM image shown in Figure 3d, a nano-size columnar structure was not clearly visible on the primary-structure surface. Figure 3j shows an AFM 3D view in the 500-nm-square range. Thus, structural analyses of the fabricated Cu, Cu_2_O, and CuO thin films on PP substrates indicated light transparency and nano-size columns on their surface. Among them, the Cu thin film exhibited a column structure with maximum sharpness and height.

### Surface Oxidization States of the Fabricated Thin Films

**Figure 4:**
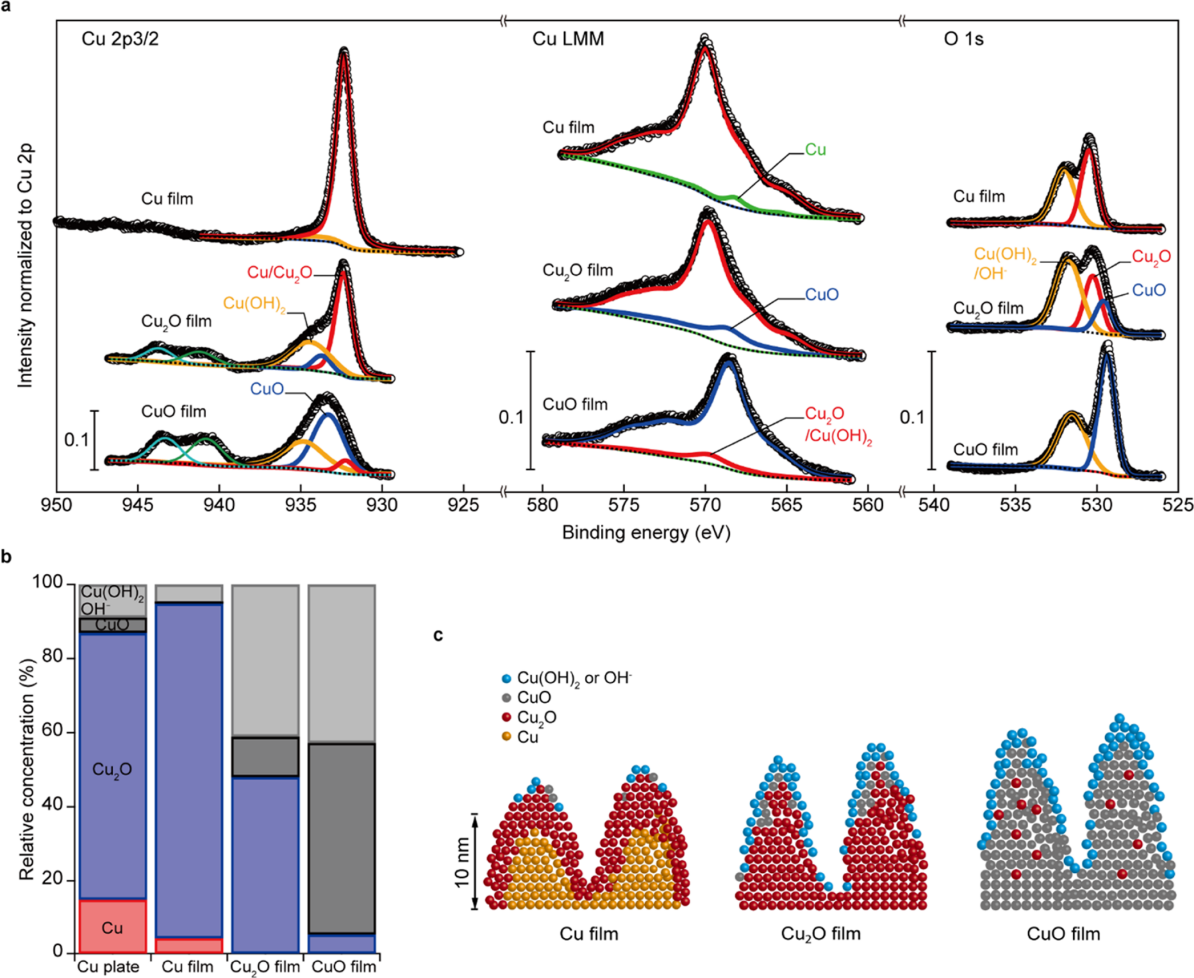
Initial surface oxidization states of the fabricated pristine Cu and Cu oxide thin films. (a) Cu 2p_3/2_, Cu LMM, and O 1s regions of the XPS spectra of pristine (with air exposure for less than 30 min) thin films. The value of 0.1 is shown in each region for the scale. The measured data points are indicated by open circles. The Shirley-type background is shown by black dotted lines, with the composite function (which is the combination of fitting functions) shown in black lines. Red lines in the Cu 2p_3/2_ region indicate the Cu and Cu_2_O contributions, blue lines indicate the CuO contribution, while orange lines indicate the Cu(OH)_2_ contribution. Green and light blue lines indicate the satellite peaks attributed to divalent Cu^2+^ ions of CuO and Cu(OH)_2_, respectively. The green line in the Cu LMM region denotes the Cu-metal contribution, red lines indicate Cu_2_O and Cu(OH)_2_ contributions, and blue lines show the CuO contribution. Red and blue lines in the O 1s region show the Cu_2_O and CuO contributions, respectively, while orange lines indicate the contributions of the Cu(OH)_2_ or OH^–^ adsorbed on the copper surface. An offset was added to the intensity to improve visibility. (b) Estimated relative fractions of copper species in the fabricated pristine thin films. For comparison, the results of a commercially available Cu plate (as received) are shown on the left-hand side. (c) Vertical schematics of a proposed model of the surface oxide layer structure of the fabricated thin films. Cu, Cu_2_O, CuO, and Cu(OH)_2_ or OH^–^ adsorbed on Cu are shown in orange, red, gray, and light blue, respectively.

In the XPS survey spectra of the fabricated films (Figure S7), no additional peaks were observed, except those of Si, which originated from the Si substrate underneath the films. The Cu 2p_3/2_, Cu LMM, and O 1s regions of the XPS spectra of the pristine samples are shown in Figure 4a, with deconvoluted spectra. The positions of the deconvoluted peaks were in good agreement with the peak positions of copper, its related oxide species (such as Cu, Cu_2_O, CuO, and copper hydroxide [Cu(OH)_2_]), and hydroxyl groups (OH^–^) adsorbed on the copper surface reported in the literature.^26,27,30^ The intensity values were normalized by dividing the intensity by the value of the area of the entire Cu 2p spectrum and plotted against the binding energy. A scale of 0.1 of normalized intensity is shown in each region. The spectra have been arranged in the following order: pristine Cu thin film, Cu_2_O thin film, and CuO thin film, from the top of Figure 4a, with the measured data points indicated by open circles. The Shirley-type background is shown by black dotted lines, with the composite function (which is the combination of fitting functions) indicated by black lines. The composite functions were in good agreement with the measured spectra.

Spectral deconvolution of the Cu 2p_3/2_ region (Figure 4a, left) indicated the surface of the Cu thin film to be almost metallic Cu or Cu_2_O (red line). The amount of the CuO component (blue lines) was higher in the CuO thin film than in the Cu_2_O thin film. Furthermore, on the surface of the CuO thin film, copper was almost divalent. These oxidation states were consistent with the deconvolution results in the O 1s region (Figure 4a, right). The CuO components (blue line) were not observed in the Cu thin films, whereas the Cu_2_O and CuO thin films showed CuO components with Cu(OH)_2_ components (orange lines) or Cu-surface-adsorbed OH^–^. The spectrum deconvolution of the Cu LMM region (Figure 4a, middle) indicated the existence of a metallic Cu state (green), with no CuO state (blue) in the Cu thin film. Additionally, the major components of the Cu_2_O (red) and CuO (blue) thin films were Cu_2_O and CuO, respectively. As shown in Figure 4b, the relative fractions of copper species for each sample were estimated by the area fraction of the species in the Cu 2p_3/2_ and Cu LMM spectra. The results obtained using a commercially available Cu plate (Figure S8; as received) and the prepared samples were used for comparison (Figure 4b; bar graph on the left-hand side). On the surface of the Cu plate, 72% Cu_2_O (blue), 15% Cu (red), 9% Cu(OH)_2_ (gray), and 4% CuO (black) were observed. On the other hand, the fabricated Cu thin films contained 91% Cu_2_O, 4% Cu, and only 5% Cu(OH)_2_, confirming the absence of CuO, which was observed on the Cu plates. The Cu_2_O thin films contained 48% Cu_2_O, 41% Cu(OH)_2_, and 11% CuO. Thus, the relative fraction of Cu species on the fabricated Cu_2_O thin films was similar to that of commercially available Cu_2_O particles (Figure S9). The CuO thin film contained 52% CuO, 43% Cu(OH)_2_, and 5% Cu_2_O.

### Estimation of the Surface Oxide Layer Structure

The structures of the surface oxide thin films were analyzed. The practical-information depth is approximately 3 times the photoelectron-escape depth, as calculated by the equation *d* = λ sinθ. Using the mean free path value (λ) of 2.84 nm for Cu_2_O and a take-off angle (θ) of 45°, the 3*d* was ∼6 nm. The oxide thicknesses on the Cu thin film and the Cu plate were calculated using eqs 1–3. The surface of the Cu thin film consisted of a 6-nm thick Cu_2_O layer, with thin Cu phases underneath, while the Cu plate exhibited a native oxide layer consisting of 0.5-nm thick CuO and 3-nm thick Cu_2_O.

Due to the principle of XPS analysis employed in this study, it could not be used to determine if the oxides of CuO and Cu_2_O were in a layered or mixed state. In general, the native oxidized thin films contained Cu(OH)_2_ or adsorbed OH^–^ on the top surface, a stable CuO thin film, followed by a Cu_2_O thin film, with a Cu layer at the bottom.^31^ A structural model for the Cu, Cu_2_O, and CuO thin films on the Si substrate was proposed considering the points above, the calculated thickness of the oxide phase on the fabricated thin films, the fraction of the Cu chemical state estimated by XPS spectra deconvolution (Figure 4b), and the surface nanostructure indicated by FE-SEM (Figure S5). The vertical sections of the structural model are schematically shown in Figure 4c. The Cu layer is considered to be inside the column structure of the Cu thin film. The Cu_2_O thin film is composed mostly of Cu_2_O, with OH^–^ adsorbed with the Cu(OH)_2_ and CuO films on the surface of the column. The CuO film is composed mostly of CuO, with a small amount of Cu_2_O. Thus, based on the results of the XRD, FE-SEM, AFM, and XPS analyses, the initial state of the crystal phase, structure, and surface chemical states of the thin films were clearly defined.

### Bacteriophage Inactivation Test

**Figure 5:**
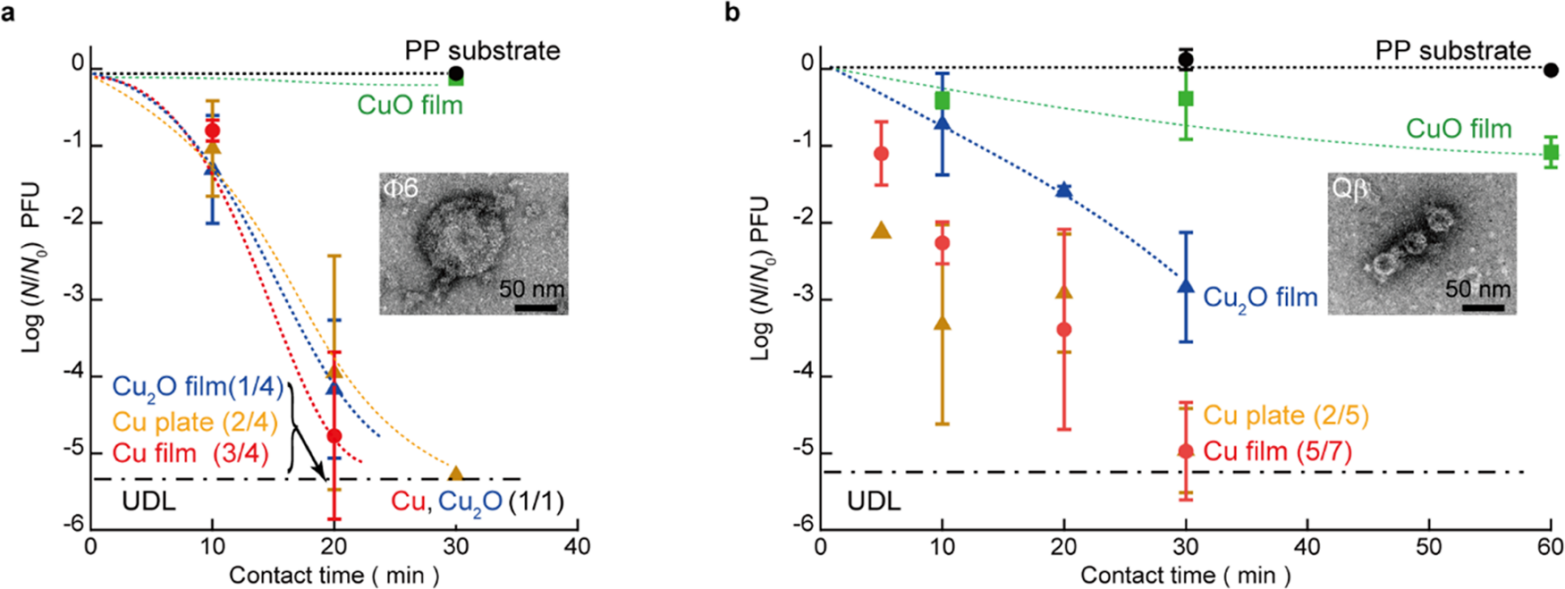
Antiviral activities against the bacteriophages Φ6 and Qβ. Time course of the log reduction of the viral titer for the bacteriophages (a) Φ6 and (b) Qβ on the fabricated thin film surfaces, as well as Cu plates and PP substrate (controls). The frequency of the case with no plaque formation (UDL: under the detection limit) is shown in parentheses. Insets show TEM images of these viruses. The dotted lines are a guide to the eye. Error bars are standard deviations. Statistical information of the data is listed in Table S3. The broken line denotes the detection limit. At measurement points where data was available both below and above the detection limit, the average values and standard deviation were calculated by uniformly setting log_10_(*N*/*N*_0_) = −5.4 for the case below the detection limit.

The antiviral activity of the fabricated Cu, Cu_2_O, and CuO thin film surfaces against two types of viruses with different surface structures (the envelope-type bacteriophage Φ6 and non-envelope-type bacteriophage Qβ) was investigated (Figure 5). The TEM images of these viruses are shown in the inset of Figure 5a,b. The diameters of Φ6 and Qβ were 90 and 30 nm, respectively. The titer value of the bacteriophage Φ6 virus rapidly decreased on contact with the Cu and Cu_2_O thin films by over 5 orders of magnitude after 30 min of contact (under the detection limit of log_10_(*N*/*N*_0_) = −5.2), whereas the CuO thin films caused no significant reduction compared to the PP substrate. This indicated that the oxidation state of copper influenced the antiviral activity. The Cu thin films, Cu plates, and Cu_2_O thin films exhibited comparable activity; a comparison of the frequency of bacteriophage Φ6 viral titers reduced below the detection limit with 20 min of contact indicated that their antiviral effect was in the following order: Cu thin films, Cu plates, and Cu_2_O thin films.

The antiviral activities for bacteriophage Qβ were similar, but clearer. The bacteriophage Qβ titer decreased by 5 orders of magnitude after 30 min on the Cu thin films and Cu plates; the log reduction of the viral titer was ∼3 and less than 1 for the Cu_2_O and CuO thin films, respectively. A comparison of the frequency of bacteriophage Qβ viral titers reduced below the detection limit with 30 min of contact indicated that the antiviral activity of the Cu thin film was higher than that of the Cu plate. Additionally, for the CuO thin film and PP substrate, the log reduction of bacteriophage Qβ viral titers was less than 1, even after 60 min of contact. The antiviral activities of the fabricated thin films against bacteriophages are summarized in Table S2.

Thus, the copper compounds exhibited significant and broad-spectrum antiviral activity, with the oxidation state of copper being a crucial factor influencing antiviral efficacy, in agreement with a previous report.^7^ Moreover, this is the first report confirming the higher antiviral activity of Cu compared to that of Cu_2_O with samples having well-defined nanostructures and surface chemical states. The fabricated Cu thin film showed similar or higher antiviral activity compared to that of the bulk Cu plate. Thus, the Cu thin films exhibited significant antiviral activity with low material consumption and a high degree of freedom (transparency and flexibility), facilitating applications. In addition, as shown in Figure S10, the most promising Cu thin films maintain their high antiviral activity despite the addition of bovine serum albumin (BSA) to the Qβ solution as a pseudo-contaminating protein from 0.01 mg mL^–1^ up to the approximate protein concentration of human saliva^35^ (1 mg mL^–1^). Therefore, fabricated Cu films can be expected to have applications in practical environments, such as viruses in saliva. The high antiviral activity of the Cu thin films could be attributed to the presence of the Cu metal component on its surface and the absence of the less active CuO (Figure 4b). The Cu thin films possibly showed slightly higher antiviral activity than the Cu plates because of their nano-column surface structure, or due to the difference in the fraction of CuO. It is necessary for the nanostructure of the thin films to be maintained during contact with the virus in order to elicit the proposed mechano-chemo-virucide action. According to the AFM observations of the fabricated Cu, Cu_2_O, and CuO thin films after 30 min of contact with the bacteriophage dispersion solvent (Figure S11), there are no significant changes in the surface nanostructures of the films. This indicates that the fabricated films are stable under plaque assay conditions.

Oxidation state analysis of the Cu thin films (Figure 4b) indicated that they contained only 5% Cu(OH)_2_, whereas the Cu_2_O thin films contained ∼40% Cu(OH)_2_. Cu(OH)_2_ undergoes facile transformation to the more stable divalent CuO.^31^ Thus, the Cu thin films containing lesser amounts of Cu(OH)_2_ are expected to show higher antiviral activity in air for longer periods of time compared to the Cu_2_O thin films. To confirm this, the relationship between the oxidation durability and antiviral activity of the Cu and Cu_2_O thin films was investigated (discussed in the next section).

### Oxidation Durability and Antiviral Activity of the Cu Thin Films

To investigate the relationship between the oxidation state and antiviral activities of the Cu, Cu_2_O, and CuO thin films, their antiviral activity after 30 min of contact against the non-envelope-type bacteriophage Qβ was investigated using samples with different exposure times to ambient air. Only the bacteriophage Qβ was used as the test virus here due to its higher stability than the envelope-type bacteriophage Φ6, which facilitates a clear antiviral activity analysis, as shown in Figure 5b. The surface oxidation states of similarly prepared samples with different air-exposure times were analyzed by XPS.

**Figure 6:**
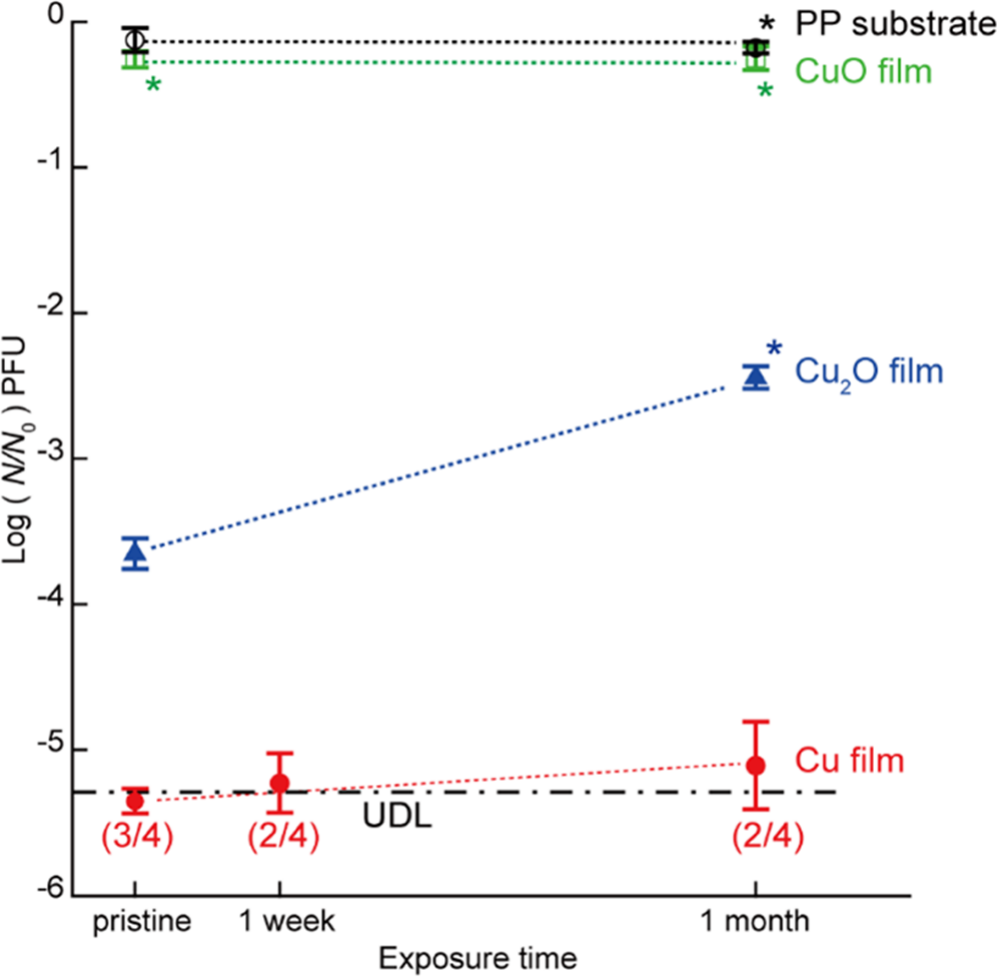
One-month antiviral activity against the bacteriophage Qβ. The log reductions of the bacteriophage Qβ viral titer on the surface of the fabricated Cu, Cu_2_O, and CuO thin films are compared after 30-min contact with the viral solution. The frequency of the case with no plaque formation (UDL: under the detection limit) is shown in parentheses. The dotted lines are a guide to the eye. Error bars are standard deviations. The number of replicate measurements of each data was three and four with and without asterisk next to the data point, respectively. The broken line denotes the lower detection limit. At measurement points where data were available both below and above the detection limit, the average values and standard deviation were calculated by uniformly setting log_10_(*N*/*N*_0_) = −5.4 for the case below the detection limit.

As shown in Figure 6, the log reduction of the bacteriophage Qβ viral titer maintained a value of less than −1 for CuO films during one-month air exposure, which is in good agreement with the results of the 30 min contact against Qβ shown in Figure 5b for pristine CuO films. This finding is consistent with the fact that the pristine CuO thin film has already formed a stable divalent (Cu^2+^) oxide film on its surface (Figure 4b). Additionally, for the PP substrate, the log reduction of bacteriophage Qβ viral titers was less than −1 during 1-month air exposure. Notably, the log reduction of the bacteriophage Qβ viral titer was −3.7 for the pristine Cu_2_O thin films; after air exposure for 1 month, the value of the log reduction was reduced by one order (to −2.4). On the other hand, the antiviral activity of the Cu thin films was stable and remained close to the detection limit during air exposure for 1 month. The number of samples under the detection limit was divided by *N* (number of samples at each point) and shown in parentheses. The log reduction of the viral titer of the pristine Cu thin film was below the lower limit of detection of −5.2 for three of the four samples, and −5.2 for one sample. Its significant antiviral activity was maintained during 1 month of air exposure; the values of log reduction of the viral titer were under the detection limit of −5.2 for half of the samples. Surprisingly, the log reduction of the viral titer by the Cu thin film was better than that of the pristine Cu_2_O thin film, even after 1 month of air exposure. The red dotted line, a guide to the eye, indicated a slightly decreasing trend in antiviral effectiveness; however, as it is near the lower detection limit, a more detailed study is required for conclusive results.

Subsequently, the reasons for the antiviral activity retention of the Cu thin film after 1 month of air exposure and degradation of the Cu_2_O thin film activity were analyzed. For this, changes in the oxidation state of the Cu_2_O thin film after air exposure for 1 week were examined by XPS (Figure S12). The Cu 2p, Cu LMM, and O 1s region spectra indicated a growth of the CuO component, whereas no significant change was observed in the C 1s region. Unlike the Cu_2_O thin film, the CuO content of the Cu thin film did not increase during 1 week of air exposure (Figure S13). Notably, the metallic Cu component of the Cu thin film remained almost unchanged after 1 week of exposure to the atmosphere, as indicated by the Cu LMM spectrum. The relative fractions of copper species in the Cu and Cu_2_O thin films after 1-week air exposure are summarized in Figure S14. The chemical state of the surface of the Cu thin film remained almost unchanged, with 87% Cu_2_O, 5% Cu, 8% Cu(OH)_2_, and a negligible amount of CuO. In contrast, in the Cu_2_O thin film, the percentage of CuO increased from 11% to 18%, and progressive oxidation was observed. Thus, the CuO formed on the surface of Cu_2_O possibly functioned as a passivation layer for antiviral activity.

According to previous publications, CuO is rapidly formed by exposing Cu thin films to air, exhibiting saturation after 1 week of exposure.^31,36^ In general, the oxidation rate of Cu is related to the number of grain boundaries and the crystal orientation.^31,36^ However, the X-ray patterns in this study did not indicate a tendency for uniaxially oriented crystal formation in the fabricated thin films (Figure 2). The reason for the significantly lower oxidation rate of the prepared Cu thin films (to CuO) compared to that of the Cu_2_O thin films is not clear. However, there are previous reports of Cu thin films fabricated by the sputtering method exhibiting oxidation resistance;^37^ or the relatively lower fraction of surface Cu(OH)_2_, which is a metastable phase and precursor to CuO,^31^ possibly results in the low oxidation rate to CuO.

**Figure 7:**
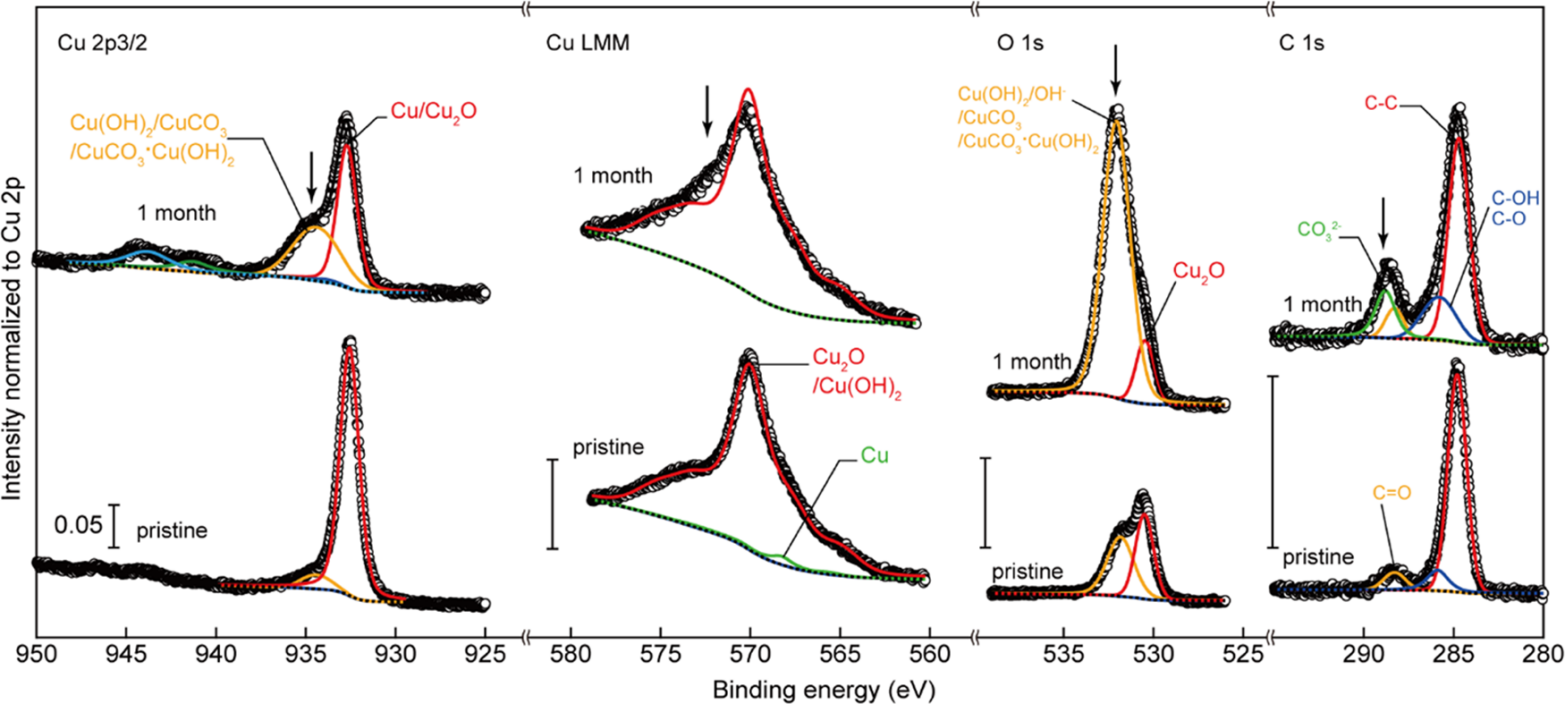
Changes in the surface oxidization state of the fabricated Cu thin film after 1 month of air exposure. The Cu 2p_3/2_, Cu LMM, O 1s, and C 1s regions of the XPS spectra of pristine, and 1-month air-exposed Cu thin films. The value of 0.05 is shown in each region for scale. The measured data points are indicated by open circles. The Shirley-type background is shown by black dotted lines, and the composite function (which is the combination of fitting functions) is shown in black lines. Red lines in the Cu 2p_3/2_ region indicate Cu and Cu_2_O, blue lines indicate CuO, and orange lines indicate Cu(OH)_2_, CuCO_3_, CuCO_3_·Cu(OH)_2_, or their total contribution. Green and light blue lines indicate satellite peaks that are attributed to divalent Cu^2+^ ions of CuO and Cu(OH)_2_. The green line in the Cu LMM region indicates the Cu-metal contribution, red lines indicate Cu_2_O and Cu(OH)_2_ contributions, and blue lines show the CuO contribution. Red and blue lines in the O 1s region show the Cu_2_O and CuO contributions, respectively, while the orange lines are related to the contributions of Cu(OH)_2_ or OH^–^ adsorbed on the copper surface. The red, blue, orange, and green lines in the C 1s indicate the C–C, C–OH, C=O bond, and carbonate ion (CO_3_^2–^) contributions, respectively.^38,39^ The arrows indicate the positions of the spectral intensities due to the formation of CuCO_3_ or CuCO_3_·Cu(OH)_2_.^38,39^ The offset was added to the intensity to improve visibility.

**Figure 8:**
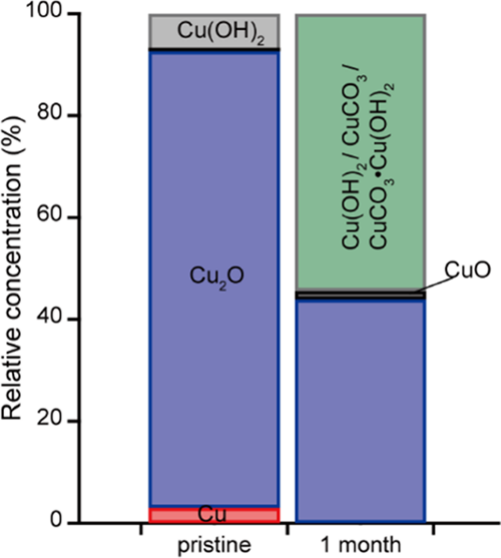
Estimated relative fractions of copper species for the fabricated pristine and 1-month air-exposed thin films. The relative fractions of Cu, Cu_2_O, CuO, and Cu(OH)_2_ are shown in red, blue, black, and gray, respectively. The fractions of Cu(OH)_2_, CuCO_3_, CuCO_3_·Cu(OH)_2_, and their sum are shown in green.

The Cu thin films that showed oxidation resistance after air exposure for 1 week were subsequently investigated considering longer periods of air exposure. The XPS spectra of the pristine and 1-month air-exposed Cu thin films are shown in Figure 7. Unlike the samples exposed to air for 1 week (Figure S13), the spectrum of the sample exposed to air for 1 month indicated oxide formation. According to spectral deconvolution in the Cu LMM region, the metallic Cu component (green line) disappeared after 1 month of air exposure, indicating progressive surface oxidation. Spectral deconvolution of the C 1s region indicated the appearance of a component (indicated by a green line in the figure) that could be assigned to carbonate ions (CO_3_^2–^).^38,39^ This peak was absent in the spectrum of the sample exposed to air for 1 week (Figure S13). Simultaneously, the intensity of other regions of the XPS spectrum increased at the energies indicated by the arrows in Figure 7. In the O 1s region, the intensity increased around the cupric carbonate CuCO_3_ or CuCO_3_·Cu(OH)_2_ binding energy,^38,39^ while the main peak at 570 eV in the Cu LMM region broadened, with an increase in intensity at ∼572 eV, consistent with CuCO_3_ or CuCO_3_·Cu(OH)_2_ growth.^38,39^ Therefore, the increase in the orange peak in the Cu 2p_3/2_ region could be attributed to the growth of CuCO_3_ or CuCO_3_·Cu(OH)_2_. The relative fractions of copper species in the Cu thin films after air exposure for 1 month are summarized in Figure 8. The chemical state of the surface of the Cu thin film changed after air exposure for 1 month; the metallic Cu component (red) (with a relative fraction of 3% in the pristine condition) disappeared, and 2% CuO (black) was observed. The oxidized component of copper was found to be 54% Cu(OH)_2_, CuCO_3_, CuCO_3_·Cu(OH)_2_, or their sum (green). Notably, even after air exposure for 1 month, the Cu thin films contained only 2% CuO, which is lower than the CuO content of the pristine Cu_2_O thin films (11%). This supports the hypothesis that the formation of CuO passivates the thin film surface and degrades its antiviral activity; the formation of CuCO_3_ or CuCO_3_·Cu(OH)_2_ does not decrease the antiviral activity to a similar extent. The oxide derivatives of copper, CuCO_3_·Cu(OH)_2_, also called malachite, are formed when Cu reacts with CO_2_ and O_2_ in the atmosphere^40,41^ and exhibit antimicrobial activity.^42^ Therefore, malachite possibly exhibits higher antiviral activity than CuO and does not form a passivation layer. As described above, the surface oxidation state of copper significantly influenced its antiviral activity. The higher antiviral effect and stability of the Cu thin film compared to the Cu_2_O thin film in air for at least 1 month has immense practical utility. Although the fraction of CuCO_3_·Cu(OH)_2_ formed on the Cu-film surface among several other oxides (Cu(OH)_2_, CuCO_3_, and CuCO_3_·Cu(OH)_2_) was not clear, previous reports indicate that the formation of copper oxides depends on particle size; studies on corrosion products in distilled water indicate malachite (CuCO_3_·Cu(OH)_2_) formation only in nanoparticles (not in copper micro-particles).^41^ Thus, the nanostructure of the Cu film possibly affected its oxidization products in air. These findings could facilitate the development of new strategies for the design of durable antiviral materials using metallic and oxide materials.

### Binding Inhibition of the SARS-CoV-2 Spike Protein

**Figure 9:**
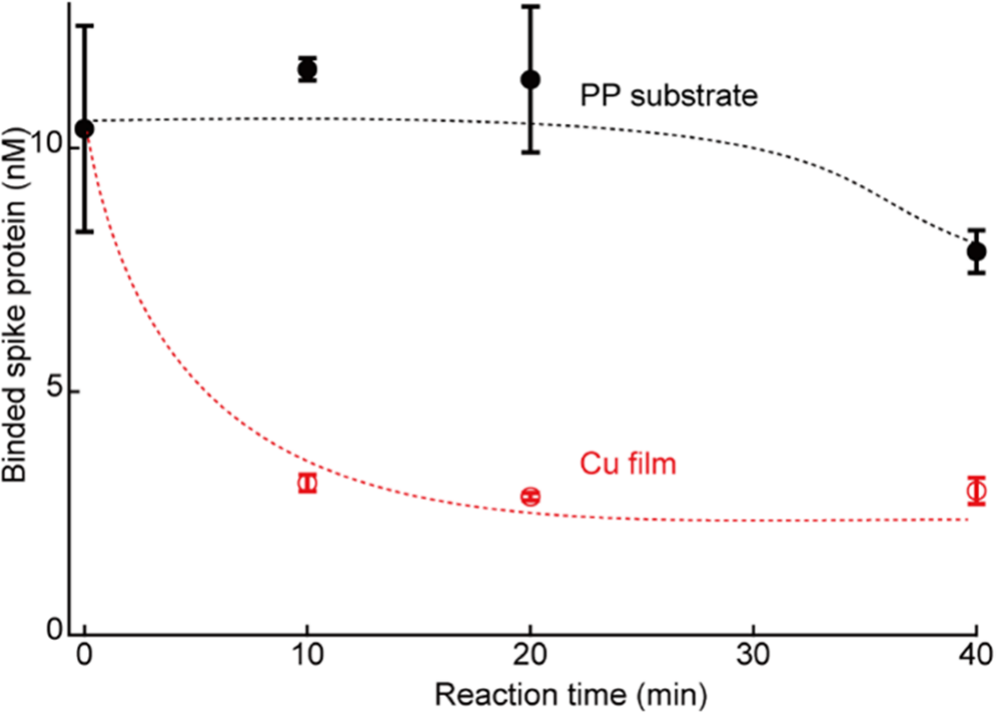
Inhibiting ability of nano-columnar Cu thin films on the binding of spike proteins to ACE2. Reductions in the concentration of spike proteins bound to ACE2 with the reaction time on the surface of the Cu thin film and PP substrate are shown. The dotted lines are a guide to the eye. Three samples have been used for each measured point and error bars are standard deviations.

Finally, the inhibiting ability of the pristine nano-columnar Cu thin films on the binding of the SARS-CoV-2 spike protein to ACE2 was investigated (Figure 9). A test solution containing 200 μL of the spike protein (10 nM) was dropped on the Cu thin film and a PP substrate (as the control) and gently shaken for different reaction times (10, 20, and 40 min). The spike protein solution dropped on the control sample (the PP substrate) did not show any inhibition of ACE2 binding for up to 40 min, whereas the Cu thin films exhibited significant ACE2-spike protein binding inhibition within 10 min. Therefore, the fabricated nano-columnar Cu thin films are expected to rapidly suppress the infection ability of SARS-CoV-2. This significant inhibiting effect could be attributed to a disulfide bond cleavage^7^ in the spike proteins by copper ions which reduces their binding affinity to ACE2.^11^ After 40-min of contact with the Cu thin film, the luminescence intensity decreased more when using a 5 nM initial concentration of spike protein than with a 10 nM concentration (Figure S15). However, the luminescence intensity did not decrease to zero even when using the 5 nM solution, suggesting that the binding ability is possibly maintained at a ratio of approximately 30%, independent of the initial concentration of the spike protein. This reduction in the spike protein:ACE2 binding ratio to 30% would correspond to an increase in the dissociation constant by approximately 2 orders of magnitude due to contact with the Cu thin film. This is consistent with previously reported results, according to which when the disulfide bonds in the RDB of the spike protein are cleaved by a reducing agent, the affinity for ACE2 drops by 2 orders of magnitude.^11^ Therefore, the results obtained in this experiment support the hypothesis that the disulfide bonds in the spike protein are cleaved by the fabricated Cu thin film, thereby inhibiting the binding of the spike protein to ACE2.

## Conclusions

In this study, nano-columnar Cu thin films, inspired by cicada wings, which exhibit mechano-bactericidal activities, were proposed. Thin films of Cu and its oxides, with a densely arranged nano-columnar structure, were fabricated by the sputtering method. This densely arranged nano-columnar structure is expected to overcome the scale gap between bacteria and viruses, enabling the application of mechano-bactericidal activities for viruses. Among the fabricated thin films, the Cu thin films showed the highest antiviral activity against both types of viral bacteriophages analyzed (the envelope-type Φ6 and non-envelope-type Qβ). Further, it was demonstrated that the fabricated Cu thin films could be applied in practical conditions (i.e., saliva) because they maintained their activity in the presence of contaminant proteins. A detailed analysis of the surface chemical states via XPS indicated that the fabricated Cu thin films showed slower oxidation (to CuO) and superior antiviral activity compared to the Cu_2_O thin films for at least 1 month of exposure to ambient air. The significant antiviral activity and oxidation durability of the Cu thin films can have immense practical utility, while their oxidation resistance can be a characteristic property of nanostructured Cu fabricated by the sputtering method. The small mass loading of the Cu thin films (6 μg cm^–2^) ensured transparency and flexibility, with low environmental risk; this is particularly useful for coating applications on disposable masks and filters. The nano-columnar Cu thin films showed slightly superior antiviral activity compared to the bulk Cu metal. However, further research is required to confirm the proposed mechano-chemo-virucidal activity and optimize the nanostructure of the thin films. Interestingly, the antiviral activity of the thin films depended on the progress of copper oxidation to CuO or other products (such as Cu(OH)_2_, CuCO_3_, and CuCO_3_·Cu(OH)_2_). We believe that these findings on the structure and surface oxidation state of copper will not only facilitate the use of copper, which has been recognized for its antimicrobial activities since 2600 B.C., in next-generation antiviral coatings, but will also guide the development of a new strategy for the design of durable antiviral materials using metals and their oxides.

## Supporting Information Available

Standard samples used for Cu LMM measurements, FE-SEM image of the columns on cicada wings, Cu LMM spectrum of standard samples, example of counting the number of appearing plaques using a colony counter, calibration curve to estimate the concentration of spike protein, FE-SEM images of the fabricated films on Si substrate, AFM line profiles and estimated roughness parameter of *R*_z_, XPS survey spectra of pristine film samples, XPS spectrum of Cu plate before and after Ar etching, estimated fraction of Cu species of commercially available Cu_2_O particles, antiviral activities against Qβ with different protein concentrations, AFM observation of thin films before and after contact with solution, XPS spectrum of Cu_2_O thin film during air exposure for 1 week and deconvolution of the spectrum, XPS spectrum of Cu thin film during air exposure for 1 week and deconvolution of the spectrum, estimated fractions of copper species of Cu film and Cu_2_O film after air exposure for 1 week, binding inhibition test using different concentrations of spike protein solutions, summary of antiviral activity against bacteriophages, area fraction of Cu species by XPS spectrum deconvolution, summary of antiviral activity against bacteriophages, and statistical information of data (PDF)
