# Supplementary material for: Durability and Surface Oxidation States of Antiviral Nano-Columnar Copper Thin Films
Source: ACS Appl Mater Interfaces. 2023 Mar 22;15(16):20398–409. doi: 10.1021/acsami.3c01400 (PMC10141257; doi:10.1021/acsami.3c01400)
Supplement: Supplementary file 1 — am3c01400_si_001.pdf [file am3c01400_si_001.pdf]

Supporting Information

Durability and Surface Oxidation States of Antiviral Nano-Columnar Copper Thin Films

Keisuke Shigetoh, Rie Hirao, and Nobuhiro Ishida*

Toyota Central R&D Labs., Inc., 41-1 Yokomichi, Nagakute, Aichi 480-1192, Japan.

*Correspondence to Keisuke Shigetoh (kei-shigetoh@mosk.tytlabs.co.jp)

Supplementary Note 1:

Cu LMM spectra of commercially available Cu plate, Cu_2O and CuO powders were measured as standard samples to obtain the fitting functions for different valence copper Cu(0) Cu(I), and Cu(II) states (Figure S2). To obtain the metallic [Cu(0)] state, measurements were performed after the oxide film on the surface of the Cu plate was removed by Ar (Ar^+, 1.0 kV) sputtering for 20 min.

Supplementary Figures.

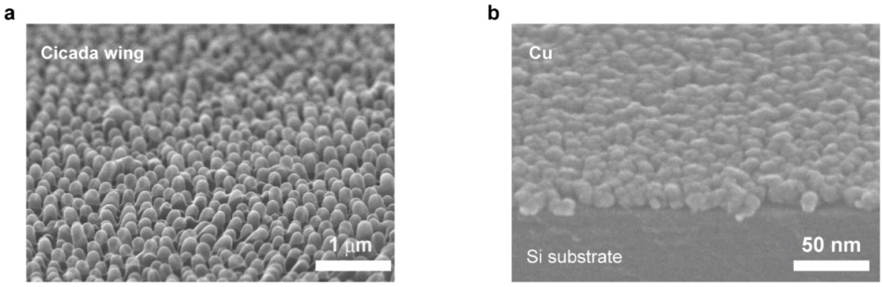

Figure S1. (a) The FE-SEM bird's-eye observation of the columns on cicada (Cryptotympana facialis) wings and (b) fabricated Cu thin film on Si substrate. Note that the scale bar is 20 times longer (1 μm) for the observed image of cicada wings than Cu thin film (50 nm).

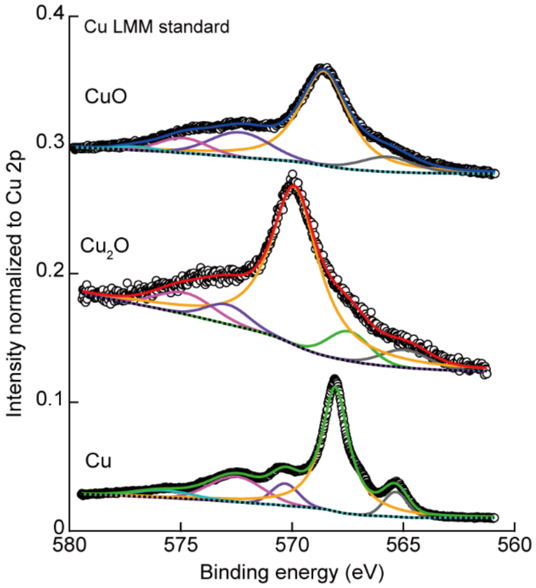

Figure S2. The Cu LMM spectrum of standard samples and their deconvolution results. The Cu LMM spectrum decomposed obtained spectrum of standard samples of CuO particles, Cu_2O particles, and Cu plate (Ar sputtered, Supplementally Note 1) by five peaks for fitting functions and obtained composite curves of Cu, Cu_2O, and CuO are shown by green, red, and blue lines, respectively.

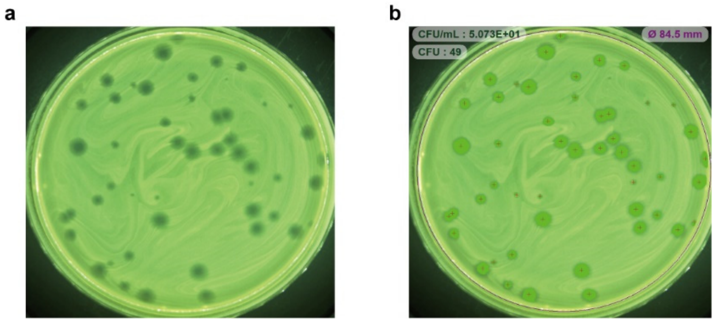

Figure S3. An example of counting the number of appearing plaques N (PFU) of bacteriophage Qβ using a colony counter. (a) Before and (b) after counting the formed plaque. The “CFU/mL” shown on top of the (b) can be ignored because the software was originally designed for colony counting. The count result was 49.

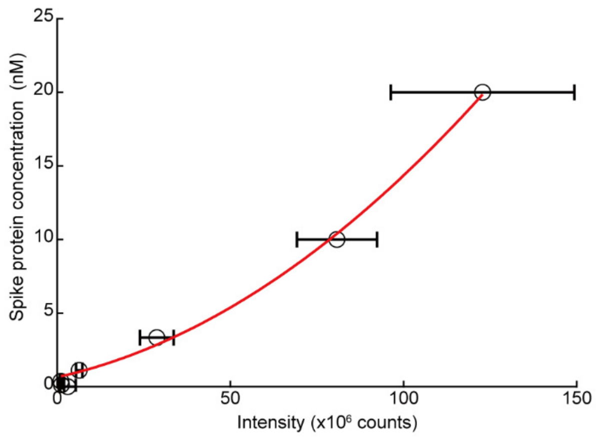

Figure S4. The calibration curve to estimate the concentration of spike S1 protein (spike protein) which was specifically bounded to ACE2. The fitting curve is shown as the red line. Three samples were used for each measured concentration of 0.12, 0.37, 1.1, 3.3, 10, and 20 nM and error bars are standard deviations.

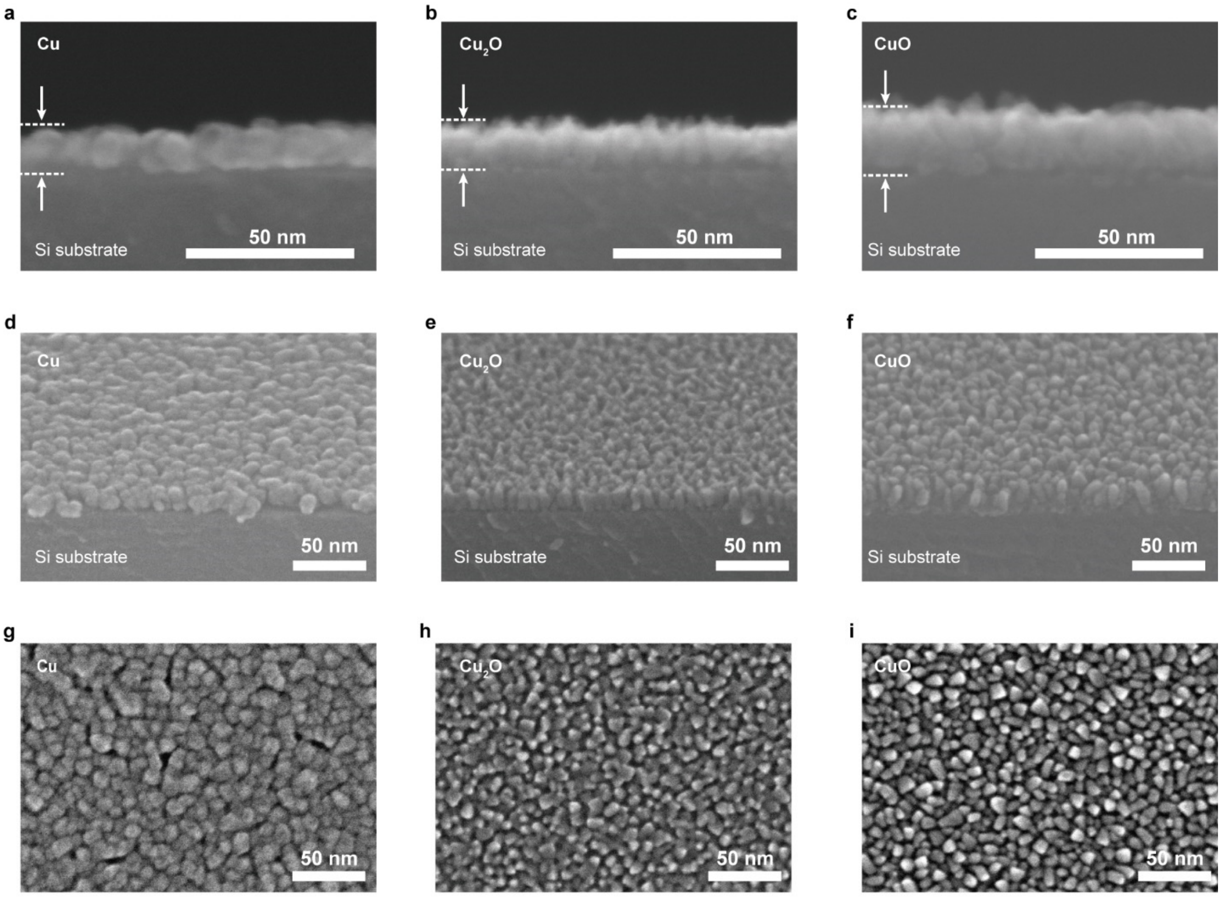

Figure S5. The FE-SEM observation of the fabricated films of Si substrate. The cross-sectional views of (a) Cu, (b) Cu_2O, and (c) CuO thin films. The arrows and dotted lines guide film thickness. The bird's-eye views of (d) Cu, (e) Cu_2O, and (f) CuO thin films. The top views of (g) Cu, (h) Cu_2O, and (i) CuO thin films.

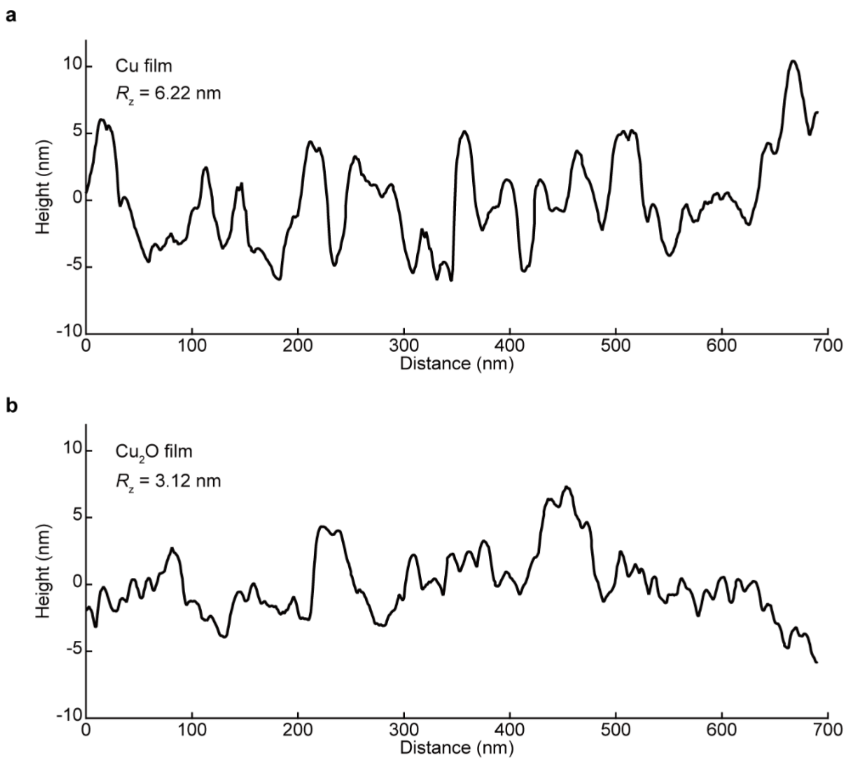

Figure S6. The line (l = 700 nm) scan results of the height by AFM and estimated one-dimensional roughness parameter Rz for the fabricated film of (a) Cu and (b) Cu_2O on polypropylene substrate.

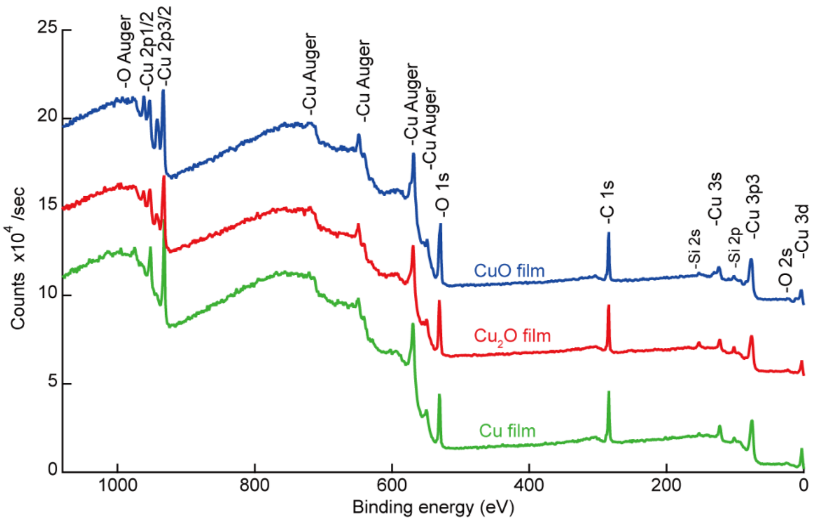

Figure S7. The XPS survey spectra of pristine film samples of fabricated Cu, Cu_2O and CuO.

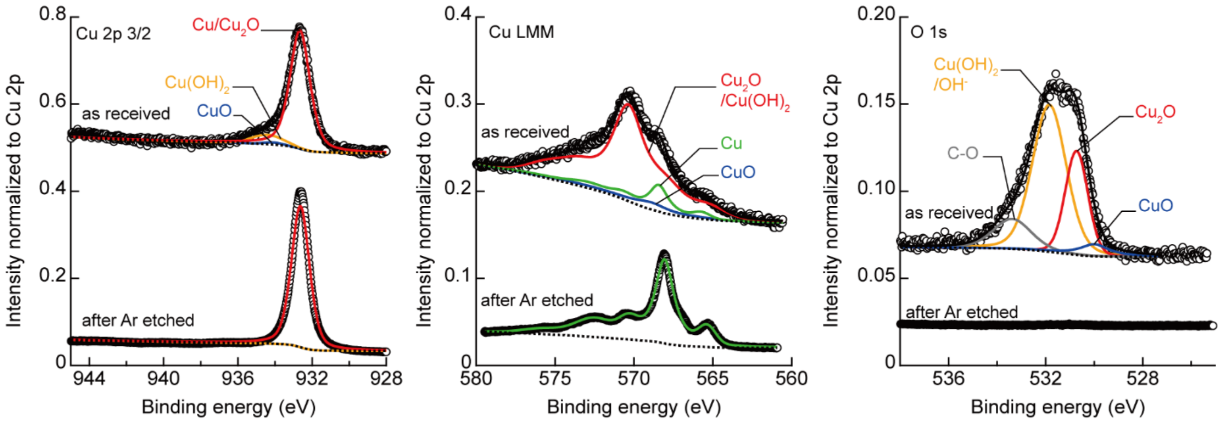

Figure S8. The XPS spectrum of Cu 2p3/2, Cu LMM, and O 1s regions for Cu plate before and after Ar etched. The measured data points are indicated by open circles. The Shirley-type background was shown by black dotted lines, and the composite function which is the combination of fitting functions is shown in black lines. Red lines in the Cu 2p3/2 region indicate Cu and Cu_2O, blue lines indicate CuO, and orange lines indicate [$Cu(OH)_2$] contributions, respectively. The green line in the Cu LMM region denotes Cu metal contribution, the red lines are Cu_2O and $Cu(OH)_2$ contributions, and the blue lines show CuO contribution. Red lines in the O 1s region show Cu_2O contribution, blue lines show CuO, orange lines are related to $Cu(OH)_2$ or OH^- adsorbed on the copper surface, and gray line are related to C-O bond. Offset was added to the intensity to improve visibility.

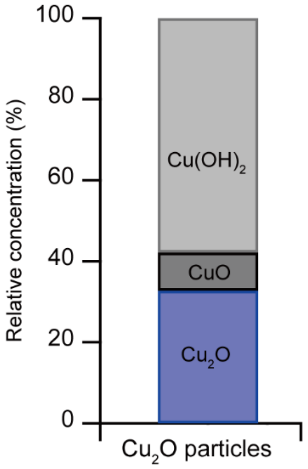

Figure S9. The estimated fraction of Cu species of commercially available Cu_2O particles (purity > 99%; 3 µm in diameter; Kojyund Chemical Laboratory) by XPS measurements was 33% Cu_2O (blue), 58% $Cu(OH)_2$ (gray), and 9% CuO (black).

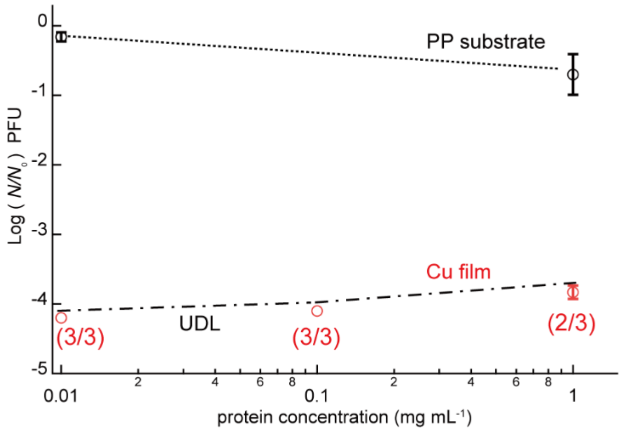

Figure S10. Antiviral activities of Cu thin film against the bacteriophage Qβ with different protein concentrations. The log reductions of the bacteriophage Qβ viral titer on the surface of the fabricated Cu thin films and PP substrate (controls) are shown compared after 30-min contact with the viral solution. The protein concentration in Qβ solution was varied up to about the protein concentration of human saliva (1 mg mL^{-1}) from 0.01 mg mL^{-1} by adding the bovine serum albumin (BSA). The frequency of the case with no plaque formation (UDL: under the detection limit) is shown in brackets in the figure. Error bars are standard deviations of the number of replicate measurements. The broken line denotes the detection limit. At measurement points where data below the detection limit were obtained, the mean value and standard deviation of the log reduction of the viral titer were calculated by uniformly setting $\log_{10}(N/N_0)$ = -4.2, -4.1, and -3.9 for the case using the viral solution that contains protein of 0.01, 0.1, and 1 mg mL^{-1}, respectively.

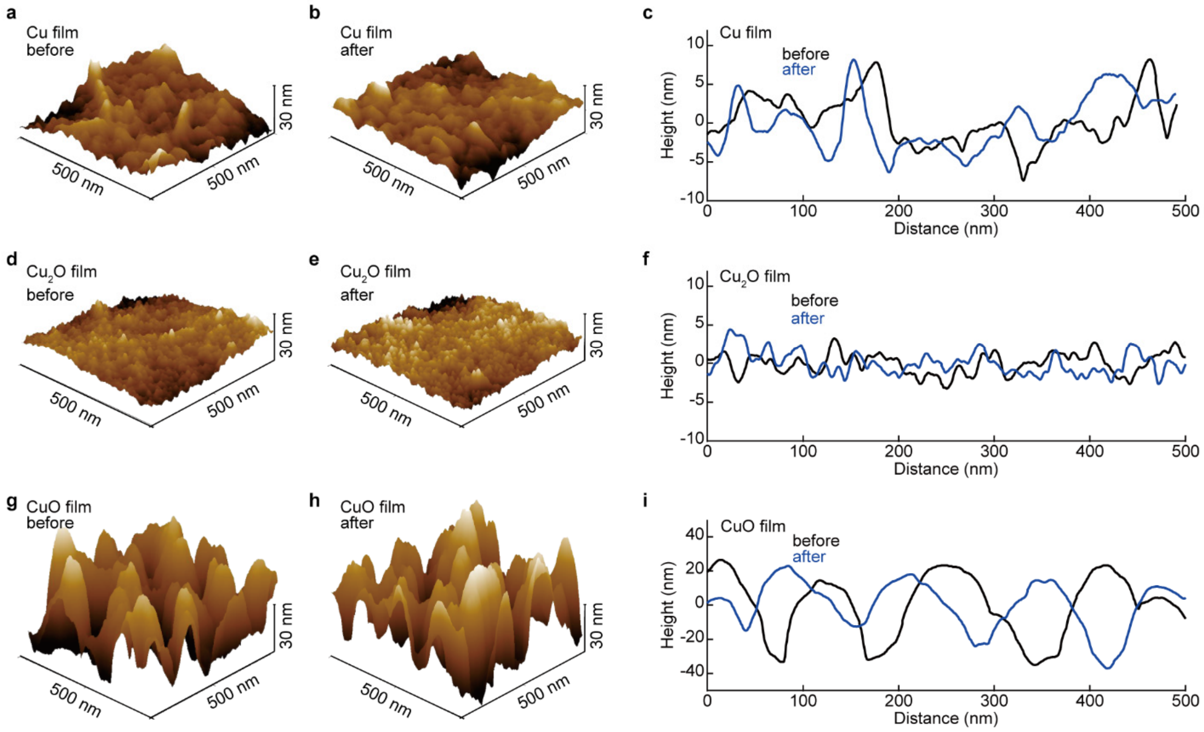

Figure S11. Stability of the surface nanostructure of the fabricated thin film in the solution. The bird's-eye view of AFM images of Cu thin films (a) before and (b) after 30 min contact with bacteriophage dispersion solvent, as well as the (c) height profiles of (black line) before and (blue line) after 30 min of contact with bacteriophage dispersion solvent. The bird's-eye view of AFM images of Cu_2O thin films (d) before and (e) after 30 min contact with bacteriophage dispersion solvent, as well as the (f) height profiles. The bird's-eye view of AFM images of CuO thin films (g) before and (h) after 30 min contact with bacteriophage dispersion solvent, as well as the (i) height profiles.

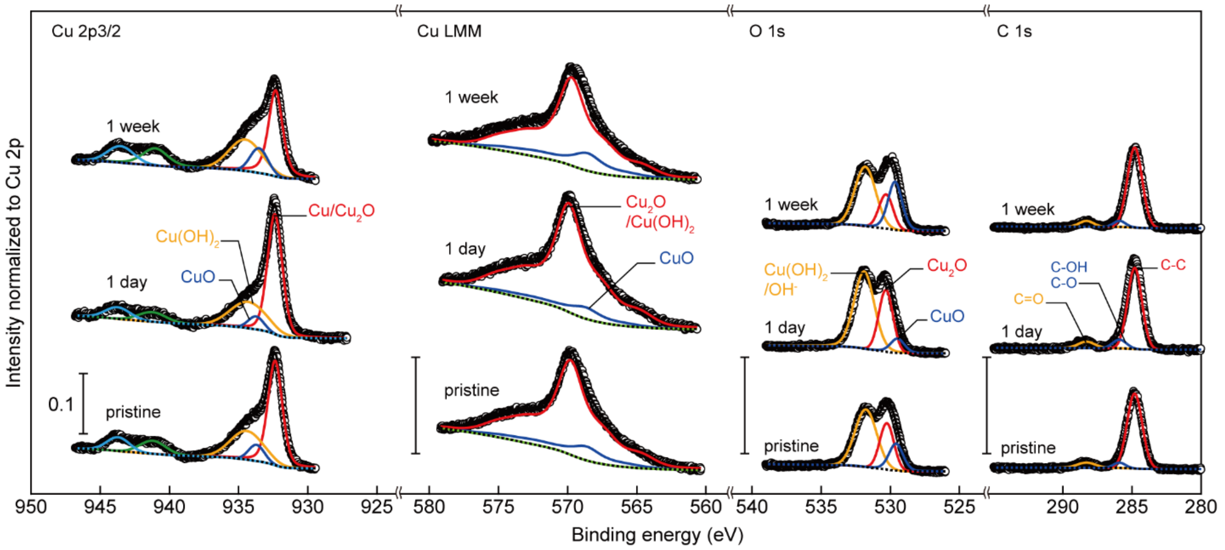

Figure S12. Changing of XPS spectrum of Cu_2O thin film during air exposer of 1 week and its deconvolution results. The Cu 2p3/2, Cu LMM, O 1s, and C 1s regions of XPS spectra of deferent air exposure time. The value of 0.1 was shown in each region for scales. The measured data points are indicated by open circles. The Shirley-type background was shown by black dotted lines, and the composite function which is the combination of fitting functions is shown in black lines. Red lines in the Cu 2p3/2 region indicate Cu and Cu_2O, blue lines indicate CuO, and orange lines indicate [$Cu(OH)_2$] contributions, respectively. Green and light blue lines are satellite peaks of are attributed to divalent Cu^{2+} ions of CuO and $Cu(OH)_2$. The green line in the Cu LMM region denotes Cu metal contribution, the red lines are Cu_2O and $Cu(OH)_2$ contributions, and the blue lines show CuO contribution. Red and blue lines in the O 1s region show the Cu_2O and CuO contributions, while the orange lines are related to the contributions of $Cu(OH)_2$ or OH^- adsorbed on the copper surface. The red, blue, and orange, lines in the C 1s indicate the C-C, C-OH, C=O bond, respectively.

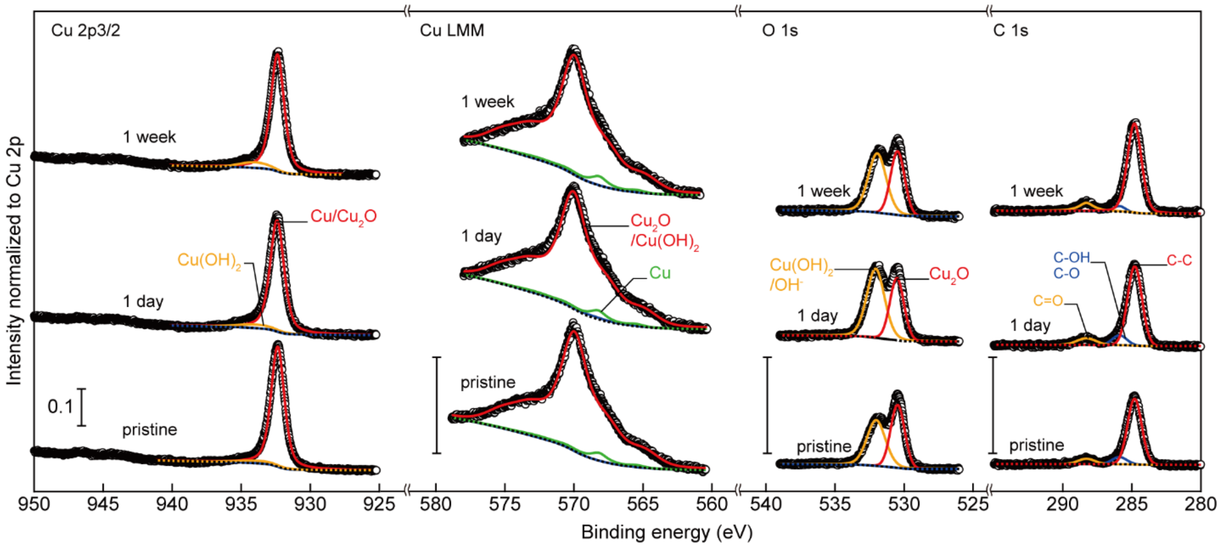

Figure S13. Changing of XPS spectrum of Cu thin film during air exposer of 1 week and its deconvolution results. The Cu 2p3/2, Cu LMM, O 1s, and C 1s regions of XPS spectra of deferent air exposure time. The value of 0.1 was shown in each region for scales. The color of the lines and the objects displayed by the symbols are the same as in Figure S10.

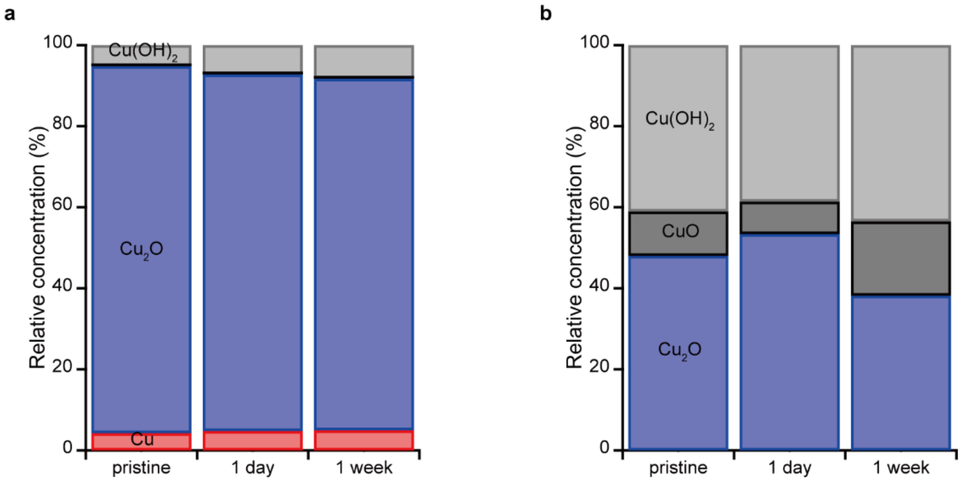

Figure S14. The changing of relative fractions of copper species for fabricated (a) Cu thin film in comparison to that of (b) Cu_2O thin film during 1-week air exposure. The relative fractions of Cu, Cu_2O, CuO, and $Cu(OH)_2$ are shown in red, blue, black, and gray, respectively.

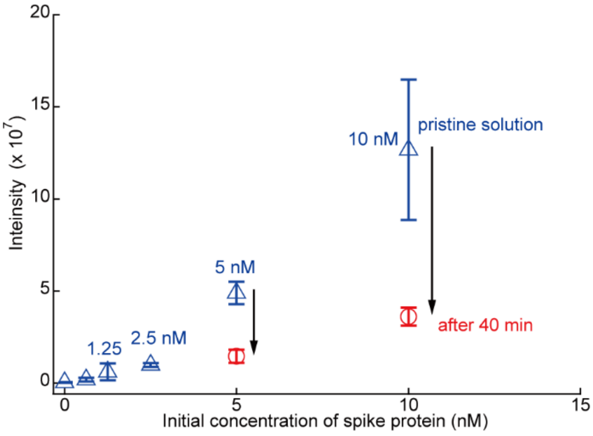

Figure S15. Inhibition ability of the binding of spike protein to ACE2 by Cu thin films against different (5 nM and 10 nM) initial concentrations of the spike protein. The luminescence intensity using pristine spike protein solution and that of after 40 min contact with the Cu thin film is shown in blue triangles and red circles, respectively. Arrows indicate the reductions of the bound amount of the spike proteins to ACE2 in 40 min contact with the Cu thin film. Three samples have been used for each measured point and error bars are standard deviations.

Supplementary Tables.

Table S1 The obtained area fraction of Cu species by XPS spectrum deconvolution.

	Cu LMM region			Cu 2p3/2 region		
	Cu	Cu_2O+$Cu(OH)_2$<br>$CuCO_3$*/$CuCO_3$·$Cu(OH)_2$*	CuO	Cu+Cu_2O	CuO	$Cu(OH)_2$<br>$CuCO_3$*/$CuCO_3$·$Cu(OH)_2$*
Area fraction %						
Cu film prisitine	4.28	95.7	0	94.8	0.04	5.14
1-week†	4.98	94.8	0.19	91.7	0.1	8.2
1-month†	0	100	0	43.9	1.6	54.5*
Cu_2O film prisitine	0	86.7	13.3	48	10.9	41.1
1-week†	0	82.7	17.3	38.2	18.3	43.5
CuO film prisitine	0	7.69	92.3	5.3	51.9	42.8
Cu plate	14.9	79.1	6.02	86.9	4.2	8.9

† Denoted the air exposure time.

* Indicates a case $CuCO_3$ or $CuCO_3$· $Cu(OH)_2$ was suggested to be formed with $Cu(OH)_2$.

Table S2 Summary of antiviral activity against bacteriophages.

	envelope-type Φ6		non-envelope-type Qβ	
	Log reduction of viral titer			
samples	20 min		30 min	
	Avg.	UDL[1]	Avg.	UDL[1]
Cu plate	- 3.9	2/4	- 5.0	2/5
Cu film	- 4.8	3/4	- 5.0	5/7
Cu_2O film	- 4.2	1/4	- 2.8	-
CuO film	- 0.1†	-	- 0.4	-

[1] Indicates a frequency of bacteriophage viral titers reduced under the detection limit. The values of the detection limit were -5.3 and -5.2 for Φ6 and Qβ, respectively.

† The result of 30 min of contact samples and viral solution.

Table S3 Statistical information of data in Figure 5.

	envelope-type Φ6						non-envelope-type Qβ					
	statistical data											
	10 min			20 min			20 min			30 min		
samples	N	Avg.	STD	N	Avg.	STD	N	Avg.	STD	N	Avg.	STD
Cu plate	3	-1.0	0.62	4	-3.9	1.5	5	-2.9	0.77	5	-5.0	0.55
Cu film	3	-0.8	0.14	4	-4.8	1.1	5	-3.4	1.3	7	-5.0	0.64
Cu_2O film	3	-1.3	0.7	4	-4.2	0.9	2	-1.6	-	8	-2.8	0.71
CuO film	-	-	-	3 †	-0.1†	0.02 †	-	-	-	5	-0.4	0.20

† The result of 30 min of contact CuO films and viral solution.
